# Synthetic cADPR analogues may form only one of two possible conformational diastereoisomers

**DOI:** 10.1038/s41598-018-33484-x

**Published:** 2018-10-15

**Authors:** Joanna M. Watt, Mark P. Thomas, Barry V. L. Potter

**Affiliations:** 1 Medicinal Chemistry and Drug Discovery, Department of Pharmacology, University of Oxford, Mansfield Road, Oxford, OX1 3QT UK; 2 Wolfson Laboratory of Medicinal Chemistry, Department of Pharmacy and Pharmacology, University of Bath, Claverton Down, Bath, BA2 7AY UK

## Abstract

Cyclic adenosine 5′-diphosphate ribose (cADPR) is an emerging Ca^2+^-mobilising second messenger. cADPR analogues have been generated as chemical biology tools via both chemo-enzymatic and total synthetic routes. Both routes rely on the cyclisation of a linear precursor to close an 18-membered macrocyclic ring. We show here that, after cyclisation, there are two possible macrocyclic product conformers that may be formed, depending on whether cyclisation occurs to the “right” or the “left” of the adenine base (as viewed along the H-8 → C-8 base axis). Molecular modelling demonstrates that these two conformers are distinct and cannot interconvert. The two conformers would present a different spatial layout of binding partners to the cADPR receptor/binding site. For chemo-enzymatically generated analogues *Aplysia californica* ADP-ribosyl cyclase acts as a template to generate solely the “right-handed” conformer and this corresponds to that of the natural messenger, as originally explored using crystallography. However, for a total synthetic analogue it is theoretically possible to generate either product, or a mixture, from a given linear precursor. Cyclisation on either face of the adenine base is broadly illustrated by the first chemical synthesis of the two enantiomers of a “southern” ribose-simplified cIDPR analogue 8-Br-*N*9-butyl-cIDPR, a cADPR analogue containing only one chiral sugar in the “northern” ribose, i.e. 8-Br-D- and its mirror image 8-Br-L-*N*9-butyl-cIDPR. By replacing the D-ribose with the unnatural L-ribose sugar, cyclisation of the linear precursor with pyrophosphate closure generates a cyclised product spectroscopically identical, but displaying equal and opposite specific rotation. These findings have implications for cADPR analogue design, synthesis and activity.

## Introduction

**Figure 1:**
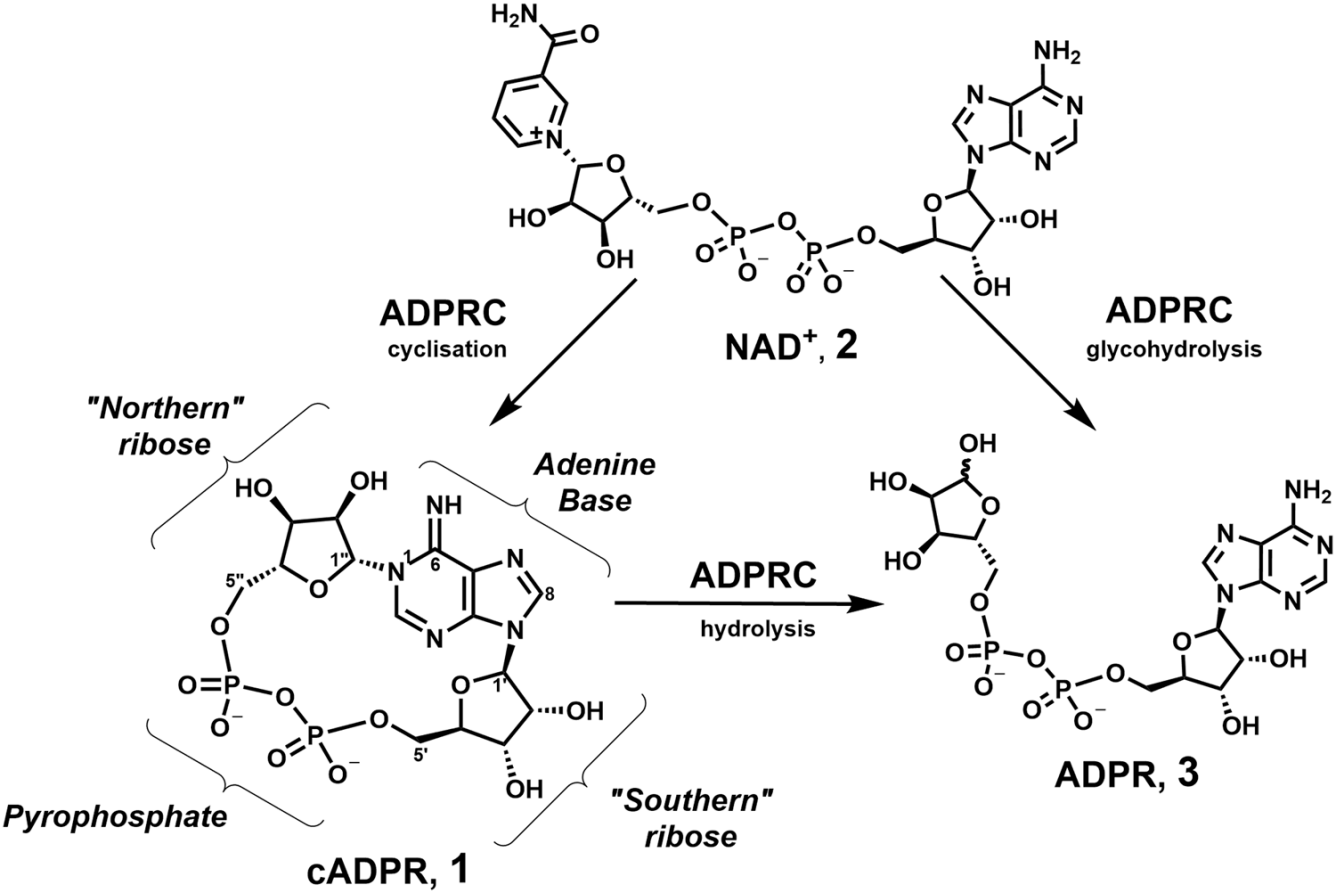
The biosynthesis of cADPR.

Cyclic adenosine 5′-diphosphate ribose (cADPR, **1**) is a cyclic dinucleotide that is produced enzymatically from nicotinamide adenine dinucleotide (NAD^+^, **2**) by ADP-ribosyl cyclases CD38 and CD157 in humans (Fig. 1)^1^. It mobilizes intracellular Ca^2+^ that in turn controls a diverse range of highly regulated cellular processes^2,3^. cADPR itself is readily hydrolysed at the labile *N*1 linkage in both neutral aqueous solution and under physiological conditions, generating another second messenger, ADPR, **3**^4,5^.

The physiological receptor for cADPR is the ryanodine receptor, although the actual Ca^2+^ release mechanism is still somewhat controversial and auxiliary cADPR binding proteins have been invoked. Recently, the apparently definitive auxiliary protein GADPH as the long-sought-after target was proposed^6^. Synthesis of stable cADPR analogues is important for studies attempting to unravel mechanistic aspects behind the cADPR/Ca^2+^ signalling system. The structure-activity relationship of cADPR-induced Ca^2+^ release revealed by cADPR analogues is still evolving and presents a complex picture. Single atomic substitutions can completely abolish activity or generate potent antagonists, and analogue activity also varies between different organisms^7^. Despite the large number of analogues prepared and the amount of pharmacological data, very little is known about whether or how these small alterations affect cADPR conformation. We previously studied the role of the “southern” ribose conformation in activity at the sea urchin cADPR receptor^7^, and a recent detailed NMR study has analysed both ribose conformations in four cADPR analogues that were prepared via the chemo-enzymatic route^8^. What now seems clear is that total synthetic routes to analogues, although labour intensive, will likely yield the most diverse structural modifications. Here, we consider an as-yet-unexplored more fundamental conformational difference that could lead to different biological activity for cADPR analogues prepared via total synthetic routes.

**Figure 2:**
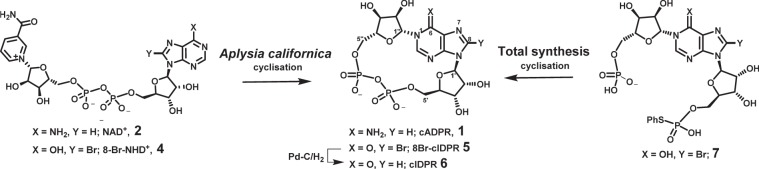
cADPR Analogue synthesis via enzyme-mediated cyclisation of NAD^+^ or via pyrophosphate formation during total synthesis.

The mechanism by which CD38 cyclises NAD^+^ to form cADPR has been studied in detail by crystallography^9–11^. Chemo-enzymatic routes to cADPR analogues rely on recognition of the relevant NAD^+^ analogue by *Aplysia californica* ADP-ribosyl cyclase. Unlike CD38, the *Aplysia californica* cyclase displays predominantly cyclase activity and is easily purified^12^. *N*1-cIDPR (**6**) was first prepared by this route and is more chemically and biologically stable than cADPR, due to a 6-NH_2_ → 6 = O substitution removing the partial positive charge from *N*1 (Fig. 2)^13^. *N*1-cIDPR acts as an agonist with equivalent potency to cADPR in permeabilised T-cells^13,14^ and 8-Br-cIDPR (**5**) was later shown to be the first membrane permeant agonist at the cADPR receptor^15^.

The main drawback of the chemo-enzymatic route is its reliance on recognition of an NAD^+^ derivative as a substrate by *Aplysia californica* ADP-ribosyl cyclase and the correct orientation of that substrate in the active site^16^. For example, in such a synthesis of cIDPR (**6**), an 8-bromo-NAD^+^ analogue (8-Br-NHD^+^, **4**) was required to generate *N*1-cIDPR (**6**) (without this 8-Br substitution, cyclisation proceeds to give the regioisomeric, fluorescent, but biologically-inactive *N*7-cIDPR)^13,14,17^. Total synthetic approaches to cADPR analogues are, however, not subject to the same limitations. In general, a linear precursor is cyclised by pyrophosphate formation, generating the macrocycle (Fig. 2). Pyrophosphate construction is frequently carried out by an intramolecular condensation reaction with an *S*-phenyl phosphorothioate in a modified Hata condensation^18^. A dilute solution of the linear precursor is treated with either I_2_ or AgNO_3_ and molecular sieves to promote intramolecular cyclisation. Total synthesis has facilitated inter alia the preparation of a number of “northern” ribose modified analogues^19–22^ that would likely be inaccessible via the chemo-enzymatic route.

cADPR itself has not been prepared via a total synthetic route. Indeed, in most reported examples, a cADPR analogue is prepared only via one of the above routes. However, we recently developed an *N*1-ribosylation methodology to introduce, stereo- and regiospecifically, a protected ribose at the *N*1 of hypoxanthine, and reported the first total synthetic route to 8-Br-cIDPR (**5**) via cyclisation of **7**^23^. The material prepared by total synthesis was spectroscopically identical to that obtained via the chemo-enzymatic route.

We report here the new idea that cADPR analogues may in principle cyclise to generate two different conformers, depending on the position of the pyrophosphate relative to the purine base. Modelling of cADPR and known analogues demonstrates that these conformers would be unable to interconvert and they would therefore exist as individual entities, with importantly potentially different biological activities. A total synthesis of the enantiomers of 8-Br-L-*N*9-butyl-cIDPR **11** and 8-Br-D-*N*9-butyl-cIDPR **9** (Fig. 8), a simplified cIDPR analogue with only one sugar moiety, illustrates in a simplified fashion to cIDPR the synthetic linear precursor cyclising through pyrophosphate formation on the two different faces of the adenine base. These two analogues represent, to the best of our knowledge, the first mirror image pair of cIDPR analogue enantiomers synthesised.

## Results

**Figure 3:**
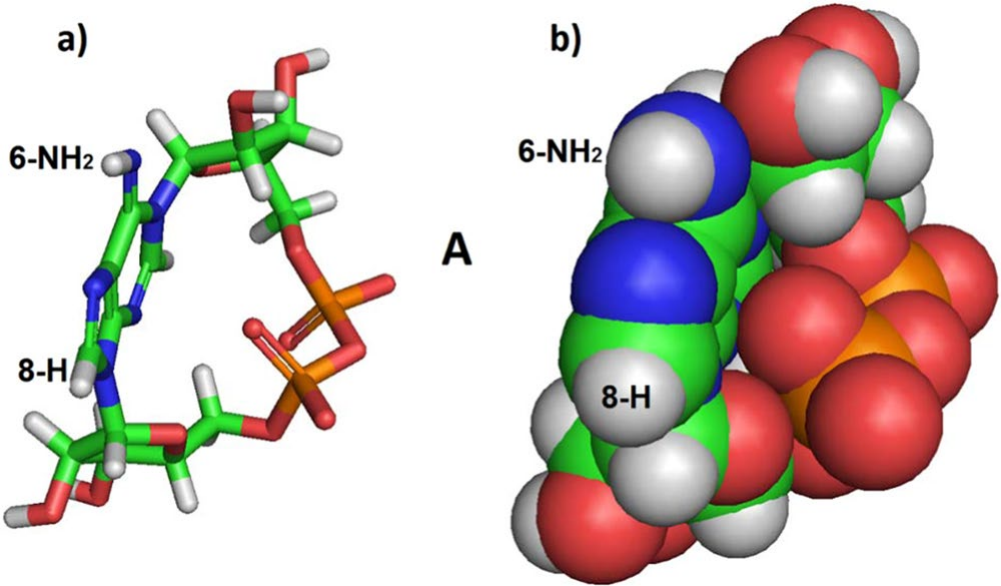
(**a**) Stick model of *N*1cADPR (**1**) conformer **A** as observed via crystallography^24^, carbons in green; (**b**) Space-filling model of the same.

The crystal structure of cADPR^24^ and the co-crystal structures of cIDPR with CD38^11^ and *Aplysia californica* ADP ribosyl cyclase^25^ all show a cyclic ligand conformation where the phosphates are located to the “right” of the purine base, when the base is orientated arbitrarily so that H-8 → C-8 is in the foreground, with H-8 pointing towards the viewer and the 6-NH_2_ or C=O respectively points upwards. Hereafter, this will be referred to as conformer **A** (Fig. 3). For easy comparison, all structures presented here are drawn with the purine base in this orientation.

**Figure 4:**
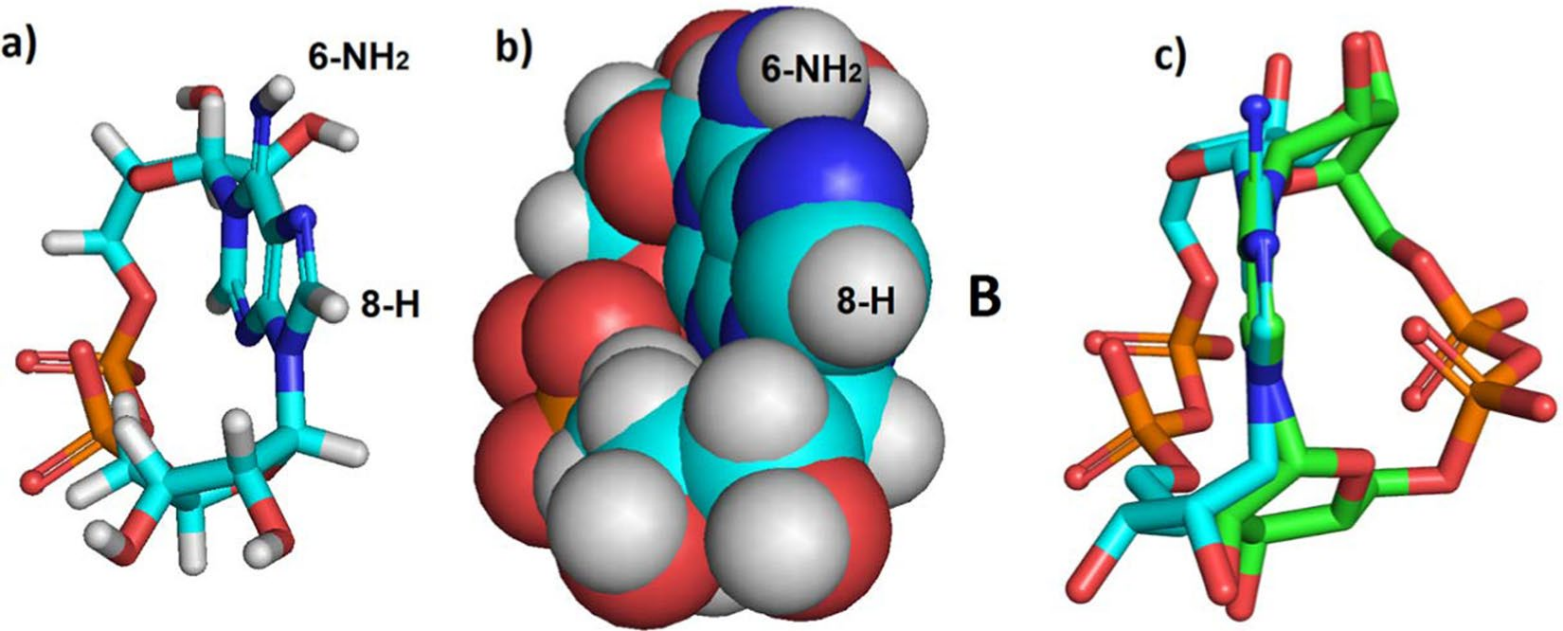
Models of proposed *N*1-cADPR (**1**) conformer **B**. (**a**) Stick representation; (**b**) Space-filling representation; (**c**) Superimposition of conformers **A** and **B** – the adenine rings are superimposed to show that cyclisation puts the phosphate groups on opposite sides of the ring. Conformer **B** shown as cyan carbons and **A** as green carbons. Note that conformers **A** and **B** are not mirror images.

Inspecting the three-dimensional structure of cADPR, we questioned whether it would also be able to adopt a second conformation (that we have termed conformer **B**) where the phosphates sit to the left of the purine base (Fig. 4a,b). If two such conformers exist then, after closure of the macrocyclic ring, there are two possibilities. Either (a) conformers **A** and **B** can interconvert and may exist in equilibrium, or settle in the most energetically favoured conformer or (b) the two conformers cannot interconvert, making them distinct entities. This latter point is important, because if the two conformers are unable to interconvert a chemical cyclisation might generate either the “unwanted” conformer **B**, or a mixture of the two conformers. This is in contrast to the enzymatic cyclisation which, presumably, invariably generates only one conformer, **A**. Based upon our current knowledge of the SAR it is only conformer **A** that is likely to have biological activity.

To model the situation, we built and minimised cADPR in both conformations, using the Schrödinger software. Conformer **A** is energetically favoured (151 Kcal mol^−1^) while conformer **B** has an energy of 161 Kcal mol^−1^. Conformer **A** was put through three rounds of dynamics simulations at increasing temperatures to determine whether the phosphates might be able to pass to the other side of the adenine ring and generate conformer **B** (Supplementary Information Files 1–3).

Conversion from conformer **A** to **B** would require the diphosphate bridge to move ‘under’ the adenine 2-H or ‘over’ the 6-NH_2_ and 8-H via simultaneous rotation around at least two bonds (those between the purine *N*1 and *N*9 and the ribose rings) as well as stretching of the bonds between the two ribose groups and distortion of the purine. At 298.15 K, only limited molecular movement is observed and there is no conversion. At 1298.15 K the molecular movement of the ribose rings and pyrophosphate is far more visible but the phosphates are still unable to move past the hydrogen atom in position 2 of the adenosine base. No conversion to conformer **B** is observed. At 4298.15 K the molecular movement is very vigorous and conversion of conformer **A** into **B** can apparently occur. This simulation was carried out five times, but in only one of them was conversion to conformer **B** observed. This suggests that a flip between conformers will occur only rarely under these extreme conditions, and presumably even more rarely (and likely not at all) under physiological conditions.

To probe whether other cADPR analogues would also have a fixed conformation, we examined conformer **A** of *N*1-cIDPR(**6**)^13^ under the same conditions (1 ps at 1298.15 K, see Supplementary Information File 4) and found that the 6-NH_2_ → 6 = O substitution did not alter the flexibility of the macrocycle. Similar substitutions in 8-Br-cADPR^26^, 6-Thio-cIDPR^27^ and 8-NH_2_-cADPR^26^ (see Supplementary Information Files 5–7) confirmed that these analogues are also trapped in conformer **A**. As might be expected, the larger 8-bromo and 8-amino substituents did not increase the possibility of conversion to conformer **B**. For completeness, we also modelled the corresponding conformer **B** of cADPR (**1**), *N*1-cIDPR (**6**), 8-Br-cADPR, 6-Thio-cIDPR, and 8-NH_2_-cADPR. These simulations confirmed the same inability to flip from conformer **B** to **A** (see Supplementary Information Files 8–12). Taken together, our results suggest that the conformer of cADPR (or cADPR analogue) that is generated during any cyclisation, chemical or enzymatic, is fixed with the phosphates trapped on one side of the purine base.

**Figure 5:**
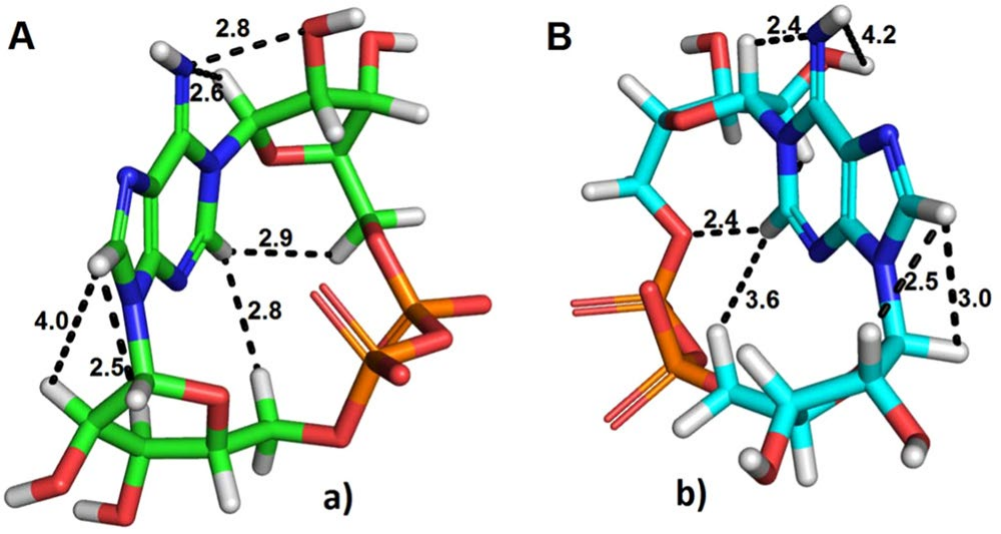
3D Shape and distances between the hydrogens of the purine ring and those of the ribose rings in both possible conformers of cADPR. (**a**) Conformer **A**, carbons in green; (**b**) conformer **B**, carbons in cyan. Distances are in Å.

Analysis of cADPR conformers **A** and **B** suggests that the adenosine and ribose-ring hydrogens would have differing nuclear environments and thus that two distinct compounds would be visible by NMR spectroscopy (Fig. 5 and Supplementary Information File 13). The “southern” ribose ring protons H-1′ and H-2′ would be affected by differing proximity to *H*-8 and the “northern” ribose *H*-1″ and *H*-2″ would be affected by their proximity to the *N*6-amino group. The changes in relative position of these groups would also likely be visible by comparison of nuclear Overhauser effect (NOE) spectra. We also measured the appropriate χ values for the sugar-base relationships of conformers **A** and **B**. Thus, in Fig. 5a the “southern” ribose-base χ angle *O*4′-*C*1′-*N*9-*C*4 is 46.8° and the northern ribose-base χ angle *O*4″-*C*1″-*N*1-*C*2 is 9.4°. In Fig. 5b the southern ribose-base χ angle *O*4′-*C*1′-*N*9-*C*4 is −6.2° and the northern ribose-base angle *O*4″-*C*1″-*N*1-*C*2 is 57.6°. All the angles fall within the range −90° to +90° and all sugar-base relationships are denoted as *syn* being thus *syn/syn* in each case. The observed χ angles are in good agreement with previous cADPR structural studies^8,24^. Conformers **A** and **B** of any analogue also possess different 3D shapes and would be expected to interact differently with the cADPR binding site. This is an important consideration for studies where biological assays are used as a readout of cADPR analogue activity, as a seemingly inactive ligand generated by total synthesis could potentially instead be the undesired and inactive conformer.

**Figure 6:**
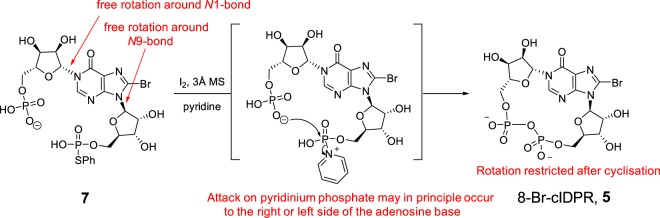
Cyclisation of a linear precursor with activated phosphate, promoted by I_2_ and molecular sieves in pyridine to generate 8-Br-cIDPR (**5**).

Therefore, during the total synthesis of a cADPR analogue – the control (or lack thereof) over which conformer is formed during cyclisation by pyrophosphate formation could either lead to the generation of a mixture of both conformers or to the undesired conformer **B**. We postulated that generation of either conformer would be theoretically possible during total synthetic routes that employ a modified Hata condensation to generate the macrocycle (Fig. 6). Since using anhydrous pyridine as a solvent is critical to this reaction^18^, activation of the mono-thiophenyl phosphate by iodine in pyridine is likely to generate the pyridinium species^28^ that is then attacked by the free phosphate to close the macrocycle. The presence of molecular sieves ensures anhydrous conditions where no activated phosphate is hydrolysed to the linear diphosphate^18^.

**Figure 7:**
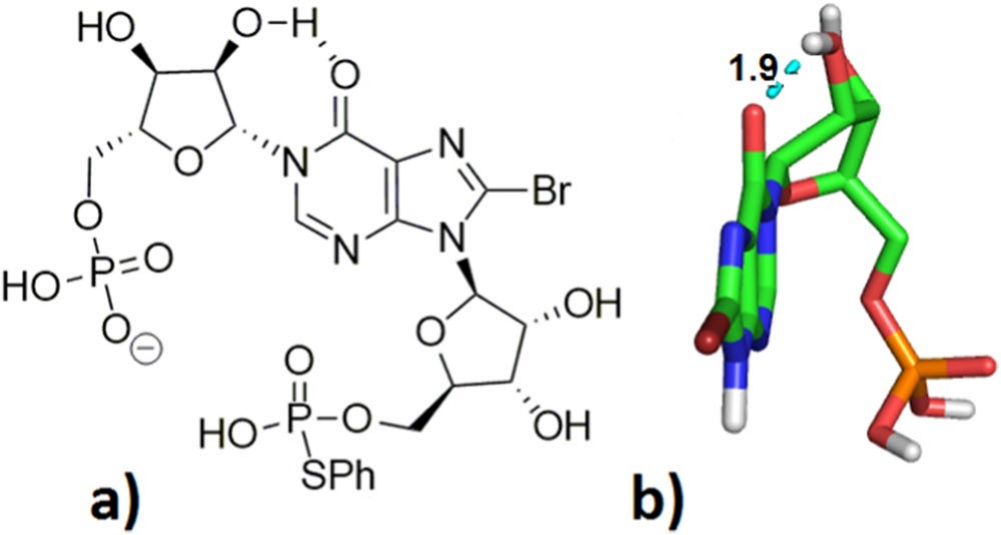
(**a**) The linear precursor to cyclisation for 8-Br-cIDPR showing a possible 6 = O → 2′-OH hydrogen bond; (**b**) minimised model of 8-Br-*N*1-inosine monophosphate demonstrates the potential for a hydrogen bond, leading to a minimum energy conformation with the “northern” phosphate positioned to the right-hand side of the base in the standard nomenclature used herein (carbons in green, hydrogen bond shown in cyan dashed line, non-polar hydrogens hidden from view).

During our total synthesis of 8-Br-cIDPR (**5**)^23^ only one product was observed during modified Hata condensation. This product was identical by ^1^H, ^13^C, ^31^P-NMR and HPLC to the 8-Br-cIDPR generated by the chemo-enzymatic route, suggesting it is conformer **A**. That only one of the two possible conformers is formed seems somewhat surprising given the flexibility of the linear precursor. Indeed, to our knowledge, there have been no reports by other groups of two conformers forming during synthetic cyclisations in cADPR analogue synthesis. However, in some instances cyclisation of a linear mono-thiophenyl phosphate precursor generated a cyclic dimer as the major product^29^. To try and rationalise the formation of only one conformer, in the light of the possibility of cyclisation occurring on either side of the adenine ring, we considered the linear precursor (**7**) to cyclisation in our intermediates that possessed an *N*1-sugar of the D-configuration (Fig. 7a). The hypoxanthine base itself is aromatic and planar, and there is rotational flexibility around both ribose C-5-phosphate groups. The ribose rings are more constrained and if there is complete free rotation around bonds *N*1, *N*9 and both ribose C-5-phosphates, it is hard to conceive the formation of only one cyclic conformer. We considered whether the *N*1 and *N*9-ribose rings could exert some control over or pre-determine the locus of pyrophosphate bond formation. For a hypoxanthine precursor such as **7** (as in Fig. 6) we postulated that an intramolecular hydrogen bond could arise between the hypoxanthine 6 = O and the 2′-OH (Fig. 7a). The resultant restriction on rotation around the *N*1-bond combined with the fixed configuration of the ribose ring may lead to a lower energy conformation with the “northern” phosphate predisposed to sit on one side of the hypoxanthine ring. Modelling of the simplified analogue, 8-Br-*N*1-inosine monophosphate, suggests a minimum energy conformation in which the 2′-OH is orientated towards the 6 = O, at a distance of 1.9 Å, a characteristic within the typical range for a hydrogen bond (Fig. 7b). This predisposes the “northern” C-5-phosphate to sit to the right of the base.

In order to illustrate the idea of cyclisation on either face of the adenine and that sugar configuration could exert a directing influence on the macrocyclisation, we wanted to carry out two separate synthetic routes to generate a pair of cADPR analogue enantiomers. Such enantiomers would have identical atomic connectivity, but be trapped with their phosphate bridges on opposite sides of the base. We chose the simplified cIDPR analogue, *N*9-butyl-cIDPR, **8**, that has only one D-ribose sugar at *N*1 and a simple butyl chain replacing the “southern” *N*9-ribose^30^. We first modelled these analogues to see if the increased flexibility afforded by the *N*9-butyl chain allowed interconversion of the two proposed macrocylic rings. In each case, analogues where either the “northern” or the “southern” ribose is replaced with a butyl chain still do not ‘flip’ from their original conformation under the standard simulation conditions. The pyrophosphate link stays on the same side of the adenine ring to the starting position (see Supplementary Information Files 14–17).

*N*9-butyl-cIDPR (**8**) has not previously been prepared via a chemo-enzymatic route. In our total synthetic route, we only observed formation of one cyclic product, prepared via 8-Br-*N*9-butyl-cIDPR (**9**). Crystallography of the 8-NH_2_-*N*9-butyl-cIDPR (**10**) complex with human CD38 (shCD38) only captured an *N*1-hydrolysed product, meaning that it was not possible to determine unequivocally on what side the cyclisation had occurred^30^. However, since the “northern” D-ribose predisposed the phosphate cyclisation to occur on the right, in cIDPR, then it is most likely that the same single product (or conformer) would be generated for *N*9-butyl-cIDPR (i.e. phosphates on the right). This is in agreement with the *N*9-butyl-cIDPR analogues being substrates for CD38, and the co-crystal structure showing that CD38 can still hydrolyse 8-NH_2_-*N*9-butyl-cIDPR (**10**)^30^.

**Figure 8:**
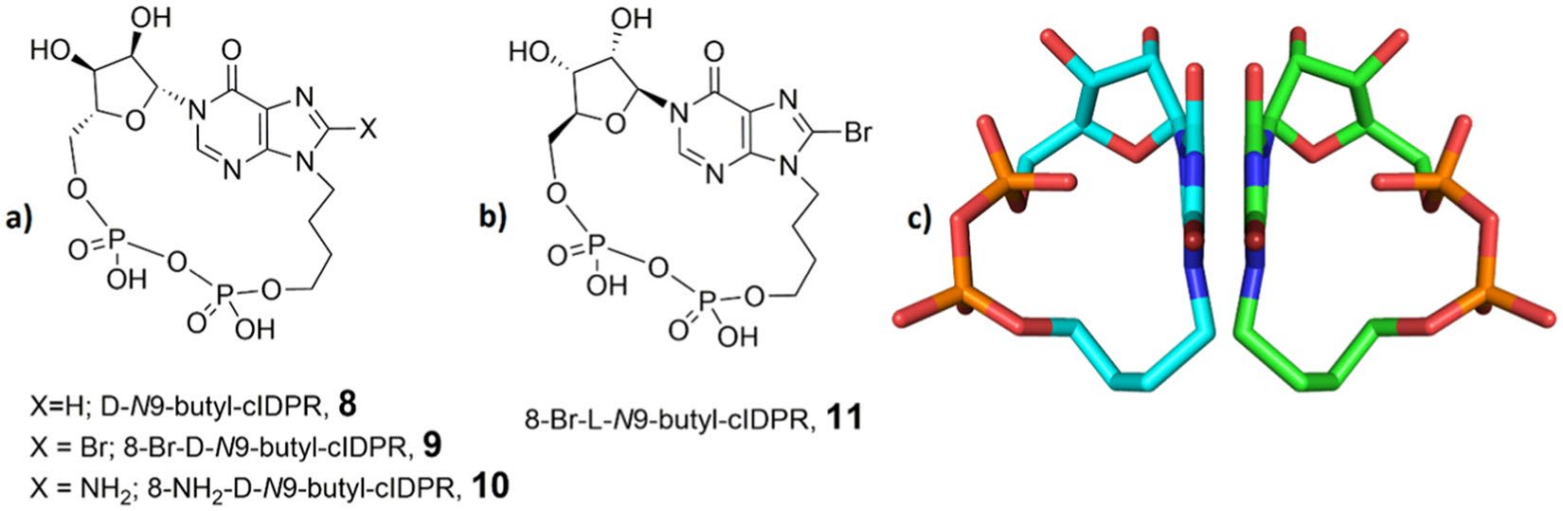
(**a**) Structure of D-*N*9-butyl-cIDPR (**8**) and precursor 8-Br-D-*N*9-butyl-cIDPR (**9**); (**b**) Structure of 8-Br-L-*N*9-butyl-cIDPR (**11**); (**c**) 8-Br-D-*N*9-butyl-cIDPR (green carbons) and 8-Br-L-*N*9-butyl-cIDPR (cyan carbons) are mirror images when cyclised. Only one potential conformer is shown in each case.

To test the idea that a linear precursor in a total synthetic route is predisposed to cyclise on one particular face, generating one of the two theoretically possible conformers, we designed a corresponding simpler and more flexible analogue with an L-configuration “northern” ribose. L-ribose, the mirror image of D-ribose, at the *N*1-position should exhibit the opposite lowest energy conformation to its D-ribose counterpart. Therefore if hydrogen bonding from the ribose 2″-OH to the hypoxanthine 6 = O were important, changing the configuration of the *N*1-ribose would predispose cyclisation to occur on the opposite face, thus generating the other enantiomer (and corresponding opposite conformer). Thus, with only one ribose sugar, cyclisation of the L-analogue to generate 8-Br-L-*N*9-butyl-cIDPR (**11**) would generate the mirror image of 8-Br-D-*N*9-butyl-cIDPR (**9**), Fig. 8.

### Synthesis of 8-Br-L-*N*9-butyl-cIDPR, 11

**Figure 9:**
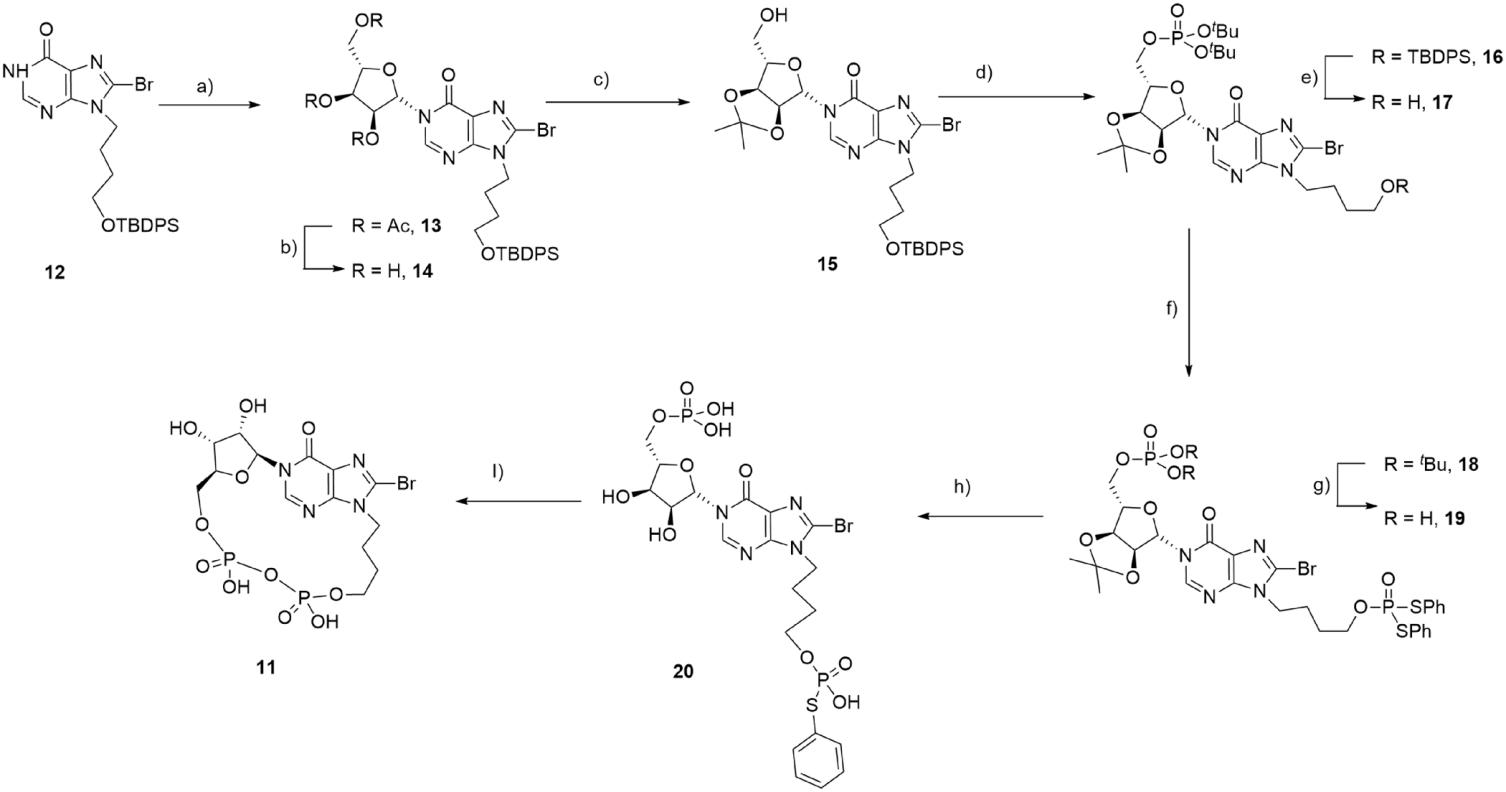
Synthesis of 8-Br-L-*N*9-butyl-cIDPR (**11**). Reagents: (**a**) (i) DBU, (ii) tetra-acetyl-L-ribose, TMSOTf, 86%; (**b**) NH_3_, MeOH, 98%; (**c**) pTsOH, H_3_CC(OMe)_2_CH_3_, Acetone, 100%; (**d**) (i) (^t^BuO)_2_PN(^i^Pr)_2_, 5-Ph-1*H*-tetrazole, DCM (ii) H_2_O_2_, Et_3_N, 80%; (**e**) TBAF.3H_2_O, AcOH, 100%; (**f**) PSS, TPS-Cl, 5-Ph-1*H*-tetrazole, pyridine, 100%; (**g**) 50% TFA (aq.), 100%; (**h**) 0.1 M NaOH-dioxane. (**i**) I_2_, 3 Å MS, pyridine, 21%.

The substrate for *N*1-ribosylation, 8-bromohypoxanthine **12**, was prepared as previously reported^30^. Deprotonation with DBU followed by addition of tetra-acetyl-L-ribose and trimethylsilyl-triflate (TMS-OTf)^23^ afforded stereo- and regioselective *N*1-coupling to generate only the desired *N*1-ribosylated product **13** in high yield. Deprotection of the three acetyl esters using methanolic ammonia generated triol **14** (Fig. 9).

The 2′,3′-diol was reprotected as an isopropylidene ketal to afford **15**, which allowed selective introduction of the first phosphate ester to the 5′-OH by treatment with *N*,*N*-diisopropyl-di-*t*-butyl-phosphoramidite and 5-phenyl-1-*H*-tetrazole as activator, followed by oxidation of the intermediate phosphite with hydrogen peroxide and triethylamine to afford **16** (Fig. 9). The silyl ether of **16** was then removed under neutral conditions to reveal the hydroxyl group. The second protected phosphate ester was introduced by treatment of the resulting **17** with cyclohexylammonium *S*,*S*-diphenylphosphorodithioate (PSS), 2,4,6-triisopropylbenzenesulfonyl chloride (TPS-Cl) and 5-phenyl-1-*H*-tetrazole in pyridine to generate the fully protected precursor for cyclisation, **18**. Sequential deprotection of the phosphates was carried out as previously described^23^ using first 50% aqueous TFA to simultaneously remove the *tert*-butyl esters and the isopropylidene ketal, then 0.1 M sodium hydroxide in dioxane, to afford **20**. Cyclization of **20** was promoted using iodine and activated 3 Å molecular sieves in pyridine^18^, under very dilute conditions, to afford the target analogue **11** (Fig. 9).

8-Br-L-*N*9-butyl-cIDPR **11** is identical by HPLC and NMR to 8-Br-D-*N*9-butyl-cIDPR, **9**. This would be expected if cyclisation of 8-Br-L-*N*9-butyl-cIDPR (**11**) had occurred on the “left” of the base, to generate the mirror image of 8-Br-D-*N*9-butyl-cIDPR (**9**) and that each enantiomer also presents as the same mirror image conformer. For the complete avoidance of doubt we measured the optical rotation of both isomers. 8-Br-L-*N*9-butyl-cIDPR (**11**) has a specific rotation of [α]^22^_D_ = +38 (MilliQ, pH = 7) and the corresponding rotation for 8-Br-D-*N*9-butyl-cIDPR (**9**) is [α]^22^_D_ = −37 (MilliQ, pH = 7). Cyclisation of **20** on the “right” of the base or a mixture of products, would also be expected to result in visible differences by NMR due to the different environments of the ribose ring protons. That only one product is generated from the linear precursor in each case suggests it is predisposed towards cyclisation on a particular face. In this example, this control presumably arises from the configuration of the “northern” ribose since the *N*9-butyl chain would be very flexible.

We recently observed surprising differences in biological activity for other cIDPR analogues prepared for the first time with an L-ribose as the “northern” ribose^31^. Thus “L-cIDPR” is a less potent inhibitor of CD38-mediated cADPR hydrolysis than cIDPR, but was surprisingly still hydrolysed by high concentrations of CD38. On the other hand, 8-Br-L-cIDPR was a potent inhibitor of CD38-mediated cADPR hydrolysis (IC_50_ = 7 µM) but was not turned over by the enzyme suggesting that it binds to CD38 in a way that is inaccessible to the catalytic machinery of the active site. Both L-ribose analogues were prepared via cyclisation of the same linear precursor and therefore must have the same conformation. While we earlier offered a robust explanation of this activity, in that the same enzymatic machinery (perhaps surprisingly) should in principle be able to cleave the new *N*1-L-ribose linkage, we now may have to consider the possibility that this synthetic compound has a conformation with the pyrophosphate on the left of the adenosine ring (i.e. conformer **B**). For these analogues, both the “northern” and “southern” ribose rings of the linear precursor may influence the location of pyrophosphate formation, relative to the adenine ring. While this consideration may not explain the differences observed between the two L-ribose analogues, it presents another possible contributing factor.

## Conclusions

Our modelling suggests that under physiological conditions *N*1-cADPR does not flip between the two possible conformers (**A** and **B**). Therefore, it could in principle exist as two distinct conformers. To our knowledge, this has not previously been taken into consideration during total synthetic routes to cADPR analogues that may generate either one (or both) possible products, with the concomitant implications for biological activity.

We have prepared 8-Br-cIDPR (**5**), using both enzyme-mediated and synthetic routes. The NMR spectrum of chemically synthesised 8-Br-cIDPR is identical to that of enzymatically synthesised material and both samples were hydrolysed by high concentrations of CD38. Taken together, this suggests that the same conformer was formed using both methods. In this case, it is perhaps fortuitous that synthetic cyclisation of the macrocycle only generates one conformer, and that a D-ribose sugar predisposes the linear compound to generate the same conformer as both the chemo-enzymatic route and the published X-ray structure of cADPR.

The first synthesis of the pair of enantiomers 8-Br-D-*N*9-butyl-cIDPR and 8-Br-L-*N*9-butyl-cIDPR reported here illustrates cyclisation on either face of the adenine. The conformational product generated may be controlled by the shape of the linear precursor.

*N*1-cADPR itself is more complex with both “northern” and “southern” D-ribose sugars. Based on our results, it seems likely that a *N*1-cADPR analogue with both L-ribose sugars prepared by total synthesis would cyclise to generate the mirror image of *N*1-cADPR with the corresponding single mirror image conformer. If one of the “northern” or “southern” ribose sugars was to be D-ribose and the other L-ribose it is less clear, however, what conformation the cyclised analogue would adopt, as it is possible that both ribose rings may affect the conformation of the linear precursor. These compounds have not yet all been synthesised, but the observations reported here may potentially contribute to the unexpected biological activity of some recent analogues we reported with a “northern” L-ribose^31^. Thus, an awareness of the possibility of different conformers and the potential implications for biological activity should be considered in any future cADPR analogue studies using total synthesis^19,21,23,31–33^. This may however be most relevant in situations where any “northern” ribose surrogate does not possess functional groups with the ability to “lock” the cyclisation precursor onto one face, as in Fig. 7, and thus control the direction of ring closure. Examples here would be cIDPRE, where the “northern” ribose is a totally flexible ether chain^34^, cIDPDE with both riboses so replaced^35^ and those similar compounds with both ether and alkane chain surrogates^22^, including more recent *N*1-pentyl and corresponding phosphonopyrophosphate analogues^33^. It could well be pertinent now to re-examine the structures and activities of all such analogues in the light of this work.

## Methods

### Synthesis

#### General

All reagents and solvents were of commercial quality and were used without further purification, unless described otherwise. Unless otherwise stated, all reactions were carried out under an inert atmosphere of argon. ^1^H, ^13^C, and ^31^P NMR spectra were collected on a Varian Mercury 400 MHz or Bruker Avance III 500 MHz Spectrometer. All ^1^H- and ^13^C-NMR assignments are based on *g*COSY, *g*HMBC, *g*HSQC, and DEPT-135 experiments. Abbreviations for splitting patterns are as follows: b, broad; s, singlet; d, doublet; t, triplet; m, multiplet etc. High resolution time-of-flight mass spectra were obtained on a Bruker Daltonics micrOTOF mass spectrometer using electrospray ionisation (ESI). Optical rotations were measured as solutions in MilliQ water (pH = 7) on a Schmidt Haensch UniPol polarimeter at the sodium D line at 22 ± 1.0 °C, in a 1 cm^3^ cell, and specific rotation is given in 10^−1^ deg cm^3^ g^−1^. Analytical HPLC analyses were carried out on a Waters 2695 Alliance module equipped with a Waters 2996 Photodiode Array Detector (210–350 nm). The chromatographic system consisted of a Hichrom Guard Column for HPLC and a Phenomenex Synergi 4μ MAX-RP 80 A column (150 × 4.60 mm), eluted at 1 mL/min with the following ion-pair buffer: 0.17% (m/v) cetrimide and 45% (v/v) phosphate buffer (pH 6.4) in MeOH. Synthetic phosphates were assayed and quantified by the Ames phosphate test.

**9-(4-*tert*-butyldiphenylsilylbutyl)-8-bromohypoxanthine (12)** was prepared as previously reported^30^.

***N*1-**(**2**′,**3**′,**5**′**-Tri-*O*-acetyl-β-L-ribofuranosyl**)**-*N*9-[4-**(***tert*-butyldiphenylsilyl**)**oxybutyl**)**-8-bromohypoxanthine** (**13**); 9-(4-*tert*-butyldiphenylsilylbutyl)-8-bromohypoxanthine (**12**, 1.0 g, 1.90 mmol) was taken up in DCM (10 mL) and DBU (854 µL, 5.71 mmol) added. After 30 minutes, 1,2,3,5-tetra-*O*-acetyl-β-L-ribofuranose (665 mg, 2.09 mmol) was added and the solution cooled to −78 °C. Trimethylsilyl trifluoromethanesulfonate (1.38 mL, 7.60 mmol) was added dropwise and the solution stirred for a further 30 min before warming to rt. After 3 h, NaHCO_3_ (sat. aq.) was added and the crude material extracted into DCM (×3). The combined organic fractions were dried (Na_2_SO_4_), and solvent evaporated under reduced pressure. The residue was purified by column chromatography on silica gel eluting with PE:EtOAc (1:0 → 1:0 v/v) to afford the *title compound* (853 mg, 57%) as a colourless glass; *R*_f_ = 0.53 (PE:EtOAc 1:3 v/v); ^1^H-NMR (400 MHz, CDCl_3_) δ 8.14 (s, 1H, 2H), 7.63 (d, 4H, *J* = 7.0), 7.40–7.34 (m, 6H) (10× Ar-H), 6.39 (d, 1H, *J* = 4.3, H-1′), 5.46 (dd, 1H, *J* = 5.6, 4.3, H-2′), 5.44 (dd, 1H, *J* = 5.6, 4.1, H-3′), 4.42–4.38 (m, 3H, H-4′ and both H-5′), 4.16 (t, 2H, *J* = 7.3, CH_2_), 3.69 (t, 2H, *J* = 5.8, CH_2_), 2.13 (s, 3H), 2.11 (s, 3H), 2.07 (s, 3H) (3× OAc), 1.97–1.90 (m, 2H, CH_2_), 1.59–1.53 (m, 2H, CH_2_), 1.02 (s, 9H) ppm; ^13^C-NMR (100 MHz, CDCl_3_) δ 170.2, 169.58, 169.57, 154.7, 148.7, 144.1, 135.5 (4C), 133.6 (2C), 129.6 (2C), 127.6 (4C), 126.1, 124.0, 87.1, 80.2, 74.2, 70.2, 63.0, 62.8, 44.6, 29.3, 26.8 (3C), 26.2, 20.8, 20.5, 20.4 19.1 ppm; HRMS (ESI^+^) calcd for C_36_H_43_N_4_O_9_Si^79^BrNa 805.1880 [(M + Na)^+^], found 805.1952, calcd for C_36_H_43_N_4_O_9_Si^81^BrNa 807.1854 [(M + Na)^+^], found 807.1889.

***N*1-**(**β-L-ribofuranosyl**)**-*N*9-[4-**(***tert*-butyldiphenylsilyl**)**oxybutyl**)**-8-bromohypoxanthine** (**14**)**;** *N*1-(2,3,5-Tri-*O*-acetyl-β-L-ribofuranosyl)-*N*9-[4-(*tert*-butyldiphenylsilyl)oxybutyl)-8-bromohypoxanthine (**13**, 850 mg, 1.09 mmol) was taken up in MeOH (10 mL) in a pressure tube. The solution was saturated with NH_3_ (g) at 0 °C, then stirred at rt for 12 h. The solvent was evaporated and the residue purified by column chromatography on silica gel eluting with DCM:Acetone (1:0 → 1:0 v/v) to afford the *title compound* (552 mg, 77%) as a white amorphous solid; *R*_f_ = 0.40 (DCM:Acetone 1:1 v/v); ^1^H-NMR (400 MHz, CDCl_3_) δ 8.46 (s, 1H, H-2), 7.62 (d, *J* = 7.8, 1.5, 4H), 7.39–7.33 (m, 6H) (10× Ar-H), 5.96 (d, 1H, *J* = 4.2, H-1′), 5.19 (br d, 1H, *J* = 3.7, OH), 4.60 (br d, 1H, *J* = 4.2, H-2′), 4.47 (br d, 1H, *J* = 3.9, H-3′), 4.43 (br s, 1H, OH), 4.23 (br s, 1H, H-4′), 4.12 (t, 2H, *J* = 7.3, CH_2_), 3.95 (d, 1H, *J* = 12.2, H-5a′), 3.85 (d, 1H, *J* = 12.2, H-5b′), 3.76 (br s, 1H, OH), 3.66 (t, 2H, *J* = 5.9, CH_2_), 1.93–1.85 (m, 2H, CH_2_), 1.56–1.50 (m, 2H, CH_2_), 1.02 (s, 9H) ppm; ^13^C-NMR (100 MHz, CDCl_3_) δ 155.9, 149.1, 145.8, 135.5 (4C), 133.6 (2C), 129.6 (2C), 127.6 (4C), 126.4, 123.7, 93.0, 85.9, 76.7, 70.3, 62.9, 61.7, 44.7, 29.3, 26.8 (3C), 26.1, 19.1 ppm; HRMS (ESI^+^) calcd for C_30_H_38_N_4_O_6_Si^79^Br 657.1744 [(M + H)^+^], found 657.1751, calcd for C_30_H_38_N_4_O_6_Si^81^Br 659.1718 [(M + H)^+^], found 659.1742.

***N*1-**(**2**′,**3**′**-*O*-isopropylidine-β-L-ribofuranosyl**)**-*N*9-[4-**(***tert*-butyldiphenylsilyl**)**oxybutyl**)**-8-bromohypoxanthine** (**15**)**;** To *N*1-(β-L-ribofuranosyl)-*N*9-[4-(*tert*-butyldiphenylsilyl)oxybutyl)-8-bromohypoxanthine (**14**, 550 mg, 0.836 mmol) in acetone:2,2-dimethoxypropane (5 mL, 4:1 v/v) was added *p*-TsOH (159 mg, 0.836 mmol). After 30 min, DCM and NaHCO_3_ (sat. aq.) were added, and the organic layer dried (Na_2_SO_4_) and all solvents evaporated. The residue was purified by column chromatography on silica gel eluting with PE:EtOAc (1:0 → 1:0 v/v) to afford the *title compound* (507 mg, 87%) as a colourless glass; *R*_f_ = 0.72 (EtOAc); ^1^H-NMR (400 MHz, CDCl_3_) δ 7.99 (s, 1H, H-2), 7.62 (dd, 4H, *J* = 7.8, 1.4), 7.41–7.34 (m, 6H) (10× Ar-H), 5.73 (d, 1H, *J* = 2.7, H-1′), 5.28 (dd, 1H, *J* = 6.4, 2.7, H-2′), 5.14 (dd, 1H, *J* = 6.4, 3.4, H-3′), 4.36 (dd, 1H, *J* = 3.4, 2.7, H-4′), 4.16 (t, 2H, *J* = 7.2, CH_2_), 3.92 (d, 1H, *J* = 12.1, H-5a′), 3.83 (ddd, 1H, *J* = 12.1, 3.6, 2.7, H-5b′), 3.68 (t, 2H, *J* = 5.9, CH_2_), 3.48 (br s, 1H, OH), 1.94–1.90 (m, 2H, CH_2_), 1.59 (s, 3H), 1.57–1.50 (m, 2H, CH_2_), 1.35 (s, 3H), 1.03 (s, 9H) ppm; ^13^C-NMR (100 MHz, CDCl_3_) δ 155.3, 149.1, 146.5, 135.5 (4C), 133.6 (2C), 129.6 (2C), 127.6 (4C), 126.5, 124.7, 114.2, 96.8, 88.1, 83.6, 80.7, 62.82, 62.79, 44.7, 29.2, 27.3, 26.8 (3C), 26.1, 25.2, 19.1 ppm; HRMS (ESI^+^) calcd for C_33_H_42_N_4_O_6_Si^79^Br 697.2052 [(M + H)^+^], found 697.2097, calcd for C_33_H_42_N_4_O_6_Si^81^Br 699.2031 [(M + H)^+^], found 699.2091.

***N*1-[2′,3′-*O*-isopropylidine-5′-*O*-(di-*tert-*butyl)-phosphoryl-β-L-ribofuranosyl]-*N*9-[4-(*tert*-butyldiphenylsilyl)oxybutyl)-8-bromohypoxanthine (16)**; To a solution of *N*1-(2′,3′-*O*-isopropylidine-β-L-ribofuranosyl)-*N*9-[4-(*tert*-butyldiphenylsilyl)oxybutyl)-8-bromohypoxanthine (15, 500 mg, 0.717 mmol) in DCM (5.0 mL) was added 5-phenyl-1-*H*-tetrazole (209 mg, 1.433 mmol) and *N*,*N-*diisopropyldi-*t-*butylphosphoramidite (339 µL, 1.076 mmol). After 2.5 h, the solution was cooled to 0 °C and Et_3_N (600 µL, 4.302 mmol) and H_2_O_2_ (157 µL, 1.792 mmol) added. The solution was allowed to warm to rt and stirred for a further 4 h, after which DCM (20 mL) and H_2_O were added. The organic layer was washed with NaHCO_3_ (sat. aq.), then brine, dried (Na_2_SO_4_) and evaporated to dryness. The residue was purified by column chromatography on silica gel eluting with PE:EtOAc (1:0 → 1:0 v/v), where both solvents contained 0.5% v/v pyridine, to afford the *title compound* (349 mg, 55%) as a colourless glass; *R*_f_ = 0.43 (PE:EtOAc 1:3 v/v); ^1^H-NMR (400 MHz, CDCl_3_) δ 8.02 (s, 1H, H-2), 7.62 (dd, 4H, *J* = 7.8, 1.5), 7.40–7.33 (m, 6H) (10× Ar-H), 5.97 (d, 1H, *J* = 1.8, H-1′), 5.08 (dd, 1H, *J* = 6.4, 1.8, H-2′), 4.99 (dd, 1H, *J* = 6.4, 4.0, H-3′), 4.42 (dd, 1H, *J* = 5.4, 4.0, H-4′), 4.27–4.13 (m, 4H, both H-5″ and CH_2_), 3.67 (t, 2H, *J* = 6.0, CH_2_), 1.95–1.88 (m, 2H, CH_2_), 1.57 (s, 3H), 1.55–1.51 (m, 2H, CH_2_), 1.44 (s, 9H), 1.43 (s, 9H), 1.33 (s, 3H), 1.02 (s, 9H) ppm; ^13^C-NMR (100 MHz, CDCl_3_) δ 154.8, 149.0, 145.9, 135.5 (4C), 133.6 (2C), 129.6 (2C), 127.6 (4C), 126.0, 124.4, 114.3, 94.3, 86.9 (d, *J* = 7.8), 85.0, 82.6 (d, 2C, *J* = 7.2), 81.5, 66.4 (d, *J* = 6.1), 62.8, 44.6, 29.8 (d, 3C, *J* = 3.2), 29.7 (d, 3C, *J* = 3.0), 29.3, 27.1, 26.8 (3C), 26.2, 25.2, 19.1 ppm; ^31^P-NMR (162 MHz, ^1^H decoupled, CDCl_3_) δ −10.2; HRMS (ESI^+^) calcd for C_41_H_58_N_4_O_9_PSi^79^BrNa 911.2786 [(M + H)^+^], found 911.2887, calcd for C_41_H_58_N_4_O_9_PSi^81^BrNa 913.2766 [(M + H)^+^], found 913.2920.

***N*1-[2**′,**3**′**-*O*-isopropylidine-5**′**-*O*-**(**di-*tert-*butyl**)**-phosphoryl-β-L-ribofuranosyl]-*N*9-**(**4-hydroxybutyl**)**-8-bromohypoxanthine** (**17**); Acetic acid (69 µL, 1.203 mmol) and TBAF.3H_2_O (362 mg, 1.146 mmol) were stirred in DMF (1.5 mL) for 30 min, after which the solution was cooled to 0 °C and *N*1-(2′,3′-*O*-isopropylidine-5′-*O*-(di-*tert-*butyl)-phosphoryl-β-L-ribofuranosyl)-*N*9-(4-hydroxybutyl)-8-bromohypo-xanthine (**16**, 340 mg, 0.382 mmol) in DMF (2.0 mL) added. The resulting solution was allowed to warm to rt and stirred for a further 4 h. The solution was diluted with ether, and NaHCO_3_ (sat. aq.) and NH_3_Cl (sat. aq.) added. The organic layer was separated, and the aqueous layer extracted with ether (3×). The combined organic layers were dried (Na_2_SO_4_), evaporated to dryness and the residue purified by column chromatography on silica gel eluting with DCM:Acetone (1:0 → 1:0 v/v), where both solvents contained 0.5% v/v pyridine, to afford the *title compound* (163 mg, 65%) as a colourless glass; *R*_f_ = 0.46 (DCM:Acetone 1:1 v/v); ^1^H-NMR (400 MHz, CDCl_3_) δ 8.08 (s, 1H, H-2), 5.99 (d, 1H, *J* = 1.7, H-1′), 5.05 (dd, 1H, *J* = 6.4, 1.7, H-2′), 4.96 (dd, 1H, *J* = 6.4, 3.9, H-3′), 4.40 (app. dd, 1H, *J* = 4.4, 4.2, H-4′), 4.25–4.11 (m, 4H, both H-5′ and CH_2_), 3.66 (t, 2H, *J* = 6.3, CH_2_), 2.45 (bs, 1H, OH), 1.89 (app. quintet, 2H, *J* = 7.2, CH_2_), 1.56 (app. quintet, 2H, *J* = 6.3, CH_2_), 1.55 (s, 3H), 1.44 (s, 9H), 1.43 (s, 9H), 1.32 (s, 3H) ppm; ^13^C-NMR (125 MHz, CDCl_3_) δ 154.8, 149.1, 145.9, 126.1, 124.4, 114.3, 94.1, 86.8 (d, *J* = 8.0), 85.2, 82.9 (d, *J* = 7.3), 82.8 (d, *J* = 7.3), 81.3, 66.5 (d, *J* = 6.3), 61.8, 44.6, 29.82 (d, 3C, *J* = 2.9), 29.80 (d, 3C, *J* = 2.9), 29.2, 27.2, 26.3, 25.3 ppm; ^31^P-NMR (162 MHz, ^1^H decoupled, CDCl_3_) δ −10.4; HRMS (ESI^+^) calcd for C_25_H_41_N_4_O_9_P^79^Br 651.1795 [(M + H)^+^], found 651.1805, calcd for C_25_H_41_N_4_O_9_P^81^Br 653.1769 [(M + H)^+^], found 653.1780.

***N*1-[2′,3′-*O*-isopropylidine-5′-*O*-(di-*tert-*butyl)-phosphoryl-β-L-ribofuranosyl]-*N*9-(4-[(diphenylthio)phosphoryl]hydroxybutyl)-8-bromohypoxanthine (18)**; *N*1-(2′,3′-*O*-isopropylidine-5′-*O*-(di-*tert-*butyl)-phosphoryl-β-L-ribofuranosyl)-*N*9-(4-hydroxybutyl)-8-bromohypoxanthine (**17**, 160 mg, 0.245 mmol) was co-evaporated from pyridine (3 × 1 mL) and taken up in pyridine (3.0 mL). This solution was added to PSS (274 mg, 0.737 mmol) which had also been co-evaporated from pyridine (3 × 1 mL). 5-Phenyl-1-*H*-tetrazole (108 mg, 0.737 mmol) and TPS-Cl (148 mg, 0.490 mmol) were added and the solution stirred at rt for 5 h. DCM and H_2_O were added, the organic layer separated and the aqueous layer washed with DCM (×2). The combined organic layer was washed with brine, dried (Na_2_SO_4_) and evaporated to dryness. The residue was purified by column chromatography on silica gel eluting with PE:EtOAc (1:0 → 1:0 v/v), where both solvents contained 0.5% v/v pyridine, to afford the *title compound* (90 mg, 40%) as a white foam; *R*_f_ = 0.71 (DCM:Acetone 1:1 v/v); ^1^H-NMR (400 MHz, CDCl_3_) δ 8.05 (s, 1H, H-2), 7.52–7.49 (m, 4H), 7.37–7.30 (m, 6H) (10× ArH), 5.98 (d, 1H, *J* = 1.8, H-1′), 5.06 (dd, 1H, *J* = 6.4, 1.8, H-2′), 4.98 (dd, 1H, *J* = 6.4, 3.9, H-3′), 4.41 (app. q, 1H, *J* = 4.3, 3.9, H-4′), 4.25–4.17 (m, 4H, both H-5″ and CH_2_), 4.11 (t, 2H, *J* = 7.2, CH_2_), 1.82 (app. quintet, 2H, *J* = 7.2, CH_2_), 1.66 (app. quintet, 2H, *J* = 6.3, CH_2_), 1.56 (s, 3H), 1.45 (s, 9H), 1.44 (s, 9H), 1.32 (s, 3H) ppm; ^13^C-NMR (100 MHz, CDCl_3_) δ 154.7, 149.0, 146.0, 135.2 (d, 4C, *J* = 5.1), 129.5 (d, 2C, *J* = 3.2), 129.4 (d, 4C, *J* = 2.5), 126.2 (d, 2C, *J* = 6.6), 125.9, 124.5, 114.3, 94.2, 86.8 (d, *J* = 7.7), 85.0, 82.7 (d, 2C, *J* = 7.2), 81.4, 67.0 (d, *J* = 8.7), 66.4 (d, *J* = 6.3), 43.9, 29.8 (d, 3C, *J* = 4.2), 29.7 (d, 3C, *J* = 4.2), 27.1, 27.0 (d, *J* = 6.8), 25.6, 25.2 ppm; ^31^P-NMR (162 MHz, ^1^H decoupled, CDCl_3_) δ 49.2, −10.2; HRMS (ESI^+^) calcd for C_37_H_49_N_4_O_10_P_2_S_2_^79^BrNa 937.1441 [(M + Na)^+^], found 937.1516, calcd for C_37_H_49_N_4_O_10_P_2_S_2_^81^BrNa 939.1420 [(M + Na)^+^], found 939.1595.

***N*1-[5**′**-*O*-phosphoryl-β-L-ribofuranosyl]-*N*9-**(**4-[**(**diphenylthio**)**phosphoryl]hydroxybutyl**)**-8-bromohypoxanthine** (**19**); *N*1-(2′,3′-*O*-isopropylidine-(5-*O*-di-*tert-*butyl)-phosphoryl-β-L-ribofuranosyl)-*N*9-(4-[(diphenylthio)phosphoryl]hydroxybutyl)-8-bromohypoxanthine (**18**, 90 mg, 0.098 mmol) was stirred in 50% TFA (4 mL) at 0 °C for 4 h. All solvents were evaporated and the residue co-evaporated with MeOH (×4). The reside was purified by column chromatography on silica gel eluting with EtOAc:MeOH:H_2_O (1:0:0 → 4:2:0 → 7:2:1 v/v/v) to afford the *title compound* (70 mg, 93%) as a colourless glass; *R*_f_ = 0.20 (EtOAc:MeOH:H_2_O 7:2:1 v/v/v); ^1^H-NMR (400 MHz, MeOD) δ 8.59 (s, 1H, H-2), 7.61–7.59 (m, 4H), 7.50–7.44 (m, 6H) (10× ArH), 6.29 (d, 1H, *J* = 3.2, H-1′), 4.43–4.25 (m, 9H, H-2′, H-3′, H-4′, both H-5′ and 2× CH_2_), 1.89 (app. quintet, 2H, *J* = 6.8, CH_2_), 1.72 (app. quintet, 2H, *J* = 5.8, CH_2_) ppm; ^13^C-NMR (100 MHz, MeOD) δ 156.9, 146.7, 136.6 (d, 4C, *J* = 5.2), 131.1 (d, 2C, *J* = 3.3), 130.8 (d, 4C, *J* = 2.7), 127.0, 126.1, 124.7, 91.2, 84.3 (d, *J* = 8.4), 76.6, 70.9, 69.2 (d, *J* = 8.8), 66.5, 45.2, 28.2 (d, *J* = 6.6), 26.8 ppm; ^31^P-NMR (161 MHz, ^1^H decoupled, MeOD) δ 50.9, 0.0 ppm; HRMS (ESI^−^) calcd for C_26_H_28_N_4_O_10_P_2_S_2_^79^Br 760.9911 [(M-H)^−^], found 760.9961, calcd for C_26_H_28_N_4_O_10_P_2_S_2_^81^Br 762.9890 [(M-H)^−^], found 762.9953.

***N*1-**(**5**′**-*O*-phosphoryl-β-L-ribofuranosyl**)**-*N*9-**(**4-[**(**diphenylthio**)**phosphoryl]hydroxybutyl**)**-8-bromohypoxanthine** (**20**)**;** *N*1-(5′-*O*-phosphate-β-L-ribofuranosyl)-*N*9-(4-[(diphenylthio)phosphoryl] hydroxybutyl)-8-bromohypoxanthine (**19**, 70 mg, 0.092 mmol) was taken up in dioxane:H_2_O (2 mL, 1:1 v/v). NaOH (300 µL, 1 M) was added and the solution stirred for 40 min at rt before addition of HCl (1 M. to pH 7). The solution was diluted with MilliQ H_2_O and washed with hexane (×3) before evaporation of all solvents to give a colourless glass which was converted to the TEA salt as described below. ^31^P-NMR (202 MHz, ^1^H decoupled, D_2_O) δ 16.8, 2.7 ppm; HRMS (ESI^−^) calcd for C_20_H_23_N_4_O_11_P_2_S^79^BrNa 690.9646 [(M + Na-2H)^−^], found 690.9694, calcd for C_20_H_23_N_4_O_11_P_2_S^81^BrNa 692.9625 [(M + Na-2H)^−^], found 692.9672.

Conversion to TEA salt: The Na^+^ salt was passed through pre-washed DOWEX H^+^ resin. Acidic fractions were neutralized with TEAB (2 mL, 1 M). All solvents were evaporated and the residue co-evaporated with H_2_O to remove excess buffer. The colourless glass obtained was used directly for cyclization.

**Cyclic-*N*9-butyl-8-bromohypoxanthine-5**′**-diphosphate L-ribose** (**L-*N*9-butyl-8-Br-cIDPR**, **11**)**;** *N*1-(5′-*O*-phosphoryl-β-L-ribofuranosyl)-*N*9-(4-[(diphenylthio)phosphoryl]hydroxybutyl)-8-bromohypoxanthine (**20**, 0.092 mmol) was evaporated from pyridine (5 mL, ×2). The residue was taken up in pyridine (40 mL) and placed in a syringe. This solution was added over 15 h to a solution of iodine (249 mg, 0.980 mmol) and activated 3 Å molecular sieves (2.0 g) in pyridine (80 mL), in the dark. The solution was filtered through celite and washed with H_2_O. After addition of TEAB (3 mL, 1 M) all solvents were evaporated, and the residue partitioned between H_2_O and CHCl_3_. The aqueous layer was washed with CHCl_3_ and evaporated to dryness. The residue was purified by semi-preparative HPLC (1.1 × 25 cm C_18_ column) eluted with acetonitrile:0.1 M TEAB (1:0 → 13:7 v/v) over 25 min. Fractions were analysed by analytical HPLC and appropriate fractions collected and evaporated under vacuum. An aromatic contaminant was removed by ion exchange chromatography on Q-sepharose (MilliQ: 1 M TEAB, 1:0 → 0:1 v/v) followed by a second reverse phase purification as detailed above to give the *title compound* (13.5 mg, 25% over 2 steps); UV (H_2_O, pH 7), λ_max_ 256 nm (ε 19,900); [α]^22^_D_ + 38 (MilliQ, pH = 7); ^1^H-NMR (500 MHz, D_2_O) δ 8.80 (s, 1H, H-2), 6.07 (d, 1H, *J* = 1.5, H-1′), 4.43–4.42 (m, 1H, H-2′), 4.37 (dd, 1H, *J* = 6.5, 4.5, H-3′), 4.33–4.28 (m, 2H), 4.26–4.15 (m, 3H), 4.09 (d, 1H, *J* = 12.0, H-5_b_″), 3.84 (br s, 1H), 1.92–1.83 (m, 2H, CH_2_), 1.41–1.38 (m, 1H, CH_a_H), 1.13–1.07 (m, 1H, CH_b_H) ppm; ^13^C-NMR (125 MHz, D_2_O) δ 156.7, 150.1, 145.0, 127.7, 122.6, 91.1, 83.5, 75.2, 68.0, 65.2, 62.8, 42.9, 24.7, 23.7 ppm; ^31^P-NMR (202 MHz, D_2_O, ^1^H-decoupled) δ −10.3 (br s), −11.3 (br s) ppm; HRMS (ESI^−^) calcd for C_14_H_18_N_4_O_11_P_2_^79^Br 558.9636 [(M-H)^−^], found 558.9632; and calcd for C_14_H_18_N_4_O_11_P_2_^81^Br 560.9616 [(M-H)^−^], found 560.9615.

### Modelling

All molecules were built using the Schrödinger software. One of the oxygens on each of the phosphate groups was given a negative charge. The newly-built compounds were minimised using the Impact module of the Schrödinger software. Molecular dynamics simulations were run using the Impact module of the Schrödinger software. Unless otherwise reported all simulations were run in a constant temperature mode, lasted for 1 ps, had an initial temperature of 298.15 K and a target temperature of 1298.15 K. Velocities were initialized from a Gaussian distribution. The OPLS_2005 force field was used with a dielectric constant of 1.0. Non-bonded cut-offs were used with the neighbour list updated every ten frames and the cut-off distance being 12 Å. The 1000 frames of the simulation each lasted 0.001 ps and every fifth frame was recorded in a trajectory file. To prevent the molecule moving around the screen during the simulation the C6 atom of the purine ring was frozen in place. All other atoms were free to move. (The temperature of 1298.15 K was used because it demonstrates a good range of movement at a rate that is not so fast that the movements are hard to observe.) Some simulations were run for 10 ps with every fifth frame from 10,000 recorded. 8-Br-*N*1-inosine monophosphate was minimised using MM2 in Chem3D. Distances between atoms in minimised ligands were measured using PyMOL.

## Electronic supplementary material

Supplementary Information

Modelling Files

## Data Availability

All data generated or analysed during this study are included in this published article and its Supplementary Information Files.
