# Supplementary material for: Synthetic cADPR analogues may form only one of two possible conformational diastereoisomers
Source: Sci Rep. 2018 Oct 15;8:15268. doi: 10.1038/s41598-018-33484-x (PMC6189198; doi:10.1038/s41598-018-33484-x)
Supplement: Supplementary file 1 — Supplementary Information [file 41598_2018_33484_MOESM1_ESM.pdf]

Synthetic cADPR analogues may form only one of two possible conformational diastereoisomers

Joanna M. Watt[1,2], Mark P. Thomas[2] and Barry V. L. Potter[1]*

1. Medicinal Chemistry and Drug Discovery, Department of Pharmacology, University of Oxford, Mansfield Road, Oxford, OX1 3QT, UK
2. Wolfson Laboratory of Medicinal Chemistry, Department of Pharmacy and Pharmacology, University of Bath, Claverton Down, Bath, BA2 7AY, UK

Contents

1. Dynamics videos/animations p2

2. ^{1}H-NMR, ^{13}C-NMR and ^{31}P-NMR p3-19

Molecular dynamics files

The dynamics can be viewed using PyMol (free download available at https://pymol.org/2/). There are hyperlinks to the appropriate files. By default they should open showing the molecules as sticks. It is a good idea to view the simulations first as sticks and then as space-filling models and to rotate the molecule around to view it from a number of angles. (Note: The size of the 10ps simulations is at the limit of what PyMol can handle: it may take a while to open, and when rotating the simulation as a space-filling model PyMol may crash. Because of this these simulations are shown as lower quality images.)

In all the figures and hyperlinked PyMol files conformer **A** is shown as green carbons and conformer **B** is shown as cyan carbons.

PyMOL files showing molecular dynamics of conformer **A** of *N*1-cADPR at 298, 1298 and 4298K, to determine if conversion between conformer **A** and conformer **B** is possible.

1. Conformer **A** 1ps at 298.15K. N1cADPR_A_Dyn298
2. Conformer **A** 1ps at 1298.15K. N1cADPR_A_Dyn1298
3. Conformer **A** 10ps at 4298.15K. N1cADPR_A_Dyn4298_10

The PyMol files showing the simulations of conformer **A** of the other *N*1-cyclised compounds are listed below. All the simulations were for 1ps at 1298.15K. They show nothing different from that observed with N1cADPR_***R*** under the same conditions.

4. *N*1-cIDPR conformer **A** 1ps at 1298.15K N1cIDPR_A_Dyn1298
5. 8-Br-*N*1-cADPR conformer **A** 1ps at 1298.15K 8BrN1cADPR_A_Dyn1298
6. 6-Thio-*N*1-cIDPR conformer **A** 1ps at 1298.15K 6-ThioN1cIDPR_A_Dyn1298
7. 8-NH_2-*N*1-cADPR conformer **A** 1ps at 1298.15K 8NH2N1cADPR_A_Dyn1298

Below are listed the PyMol files showing the simulations conformer **B** of all the above molecules. All the simulations were for 1ps at 1298.15K.

8. *N*1-cADPR conformer **B** 1ps at 1298.15K N1cADPR_B_Dyn1298
9. *N*1-cIDPR conformer **B** 1ps at 1298.15K N1cIDPR_B_Dyn1298
10. 8-Br-*N*1-cADPR conformer **B** 1ps at 1298.15K 8BrN1cADPR_B_Dyn1298
11. 6-Thio-*N*1-cIDPR conformer **B** 1ps at 1298.15K 6-ThioN1cIDPR_B_Dyn1298
12. 8-NH_2-*N*1-cADPR conformer **B** 1ps at 1298.15K 8NH2N1cADPR_B_Dyn1298

The following PyMOL file shows cADPR conformers **A** and **B** overlaid and the different environments and distances between critical hydrogen atoms.

13. Distances

Below are listed the PyMol files showing the simulations of cADPR analogues with *N*1- or *N*9-butyl chains. Both conformer **A** and conformer **B** were modelled.

14. *N*1-butyl-cADPR conformer **A** N1butylcADPR_A_Dyn1298
15. *N*1-butyl-cADPR conformer **B** N1butylcADPR_B_Dyn1298
16. *N*9-butyl-cADPR conformer **A** N9butylcADPR_A_Dyn1298
17. *N*9-butyl-cADPR conformer **B** N9butylcADPR_B_Dyn1298

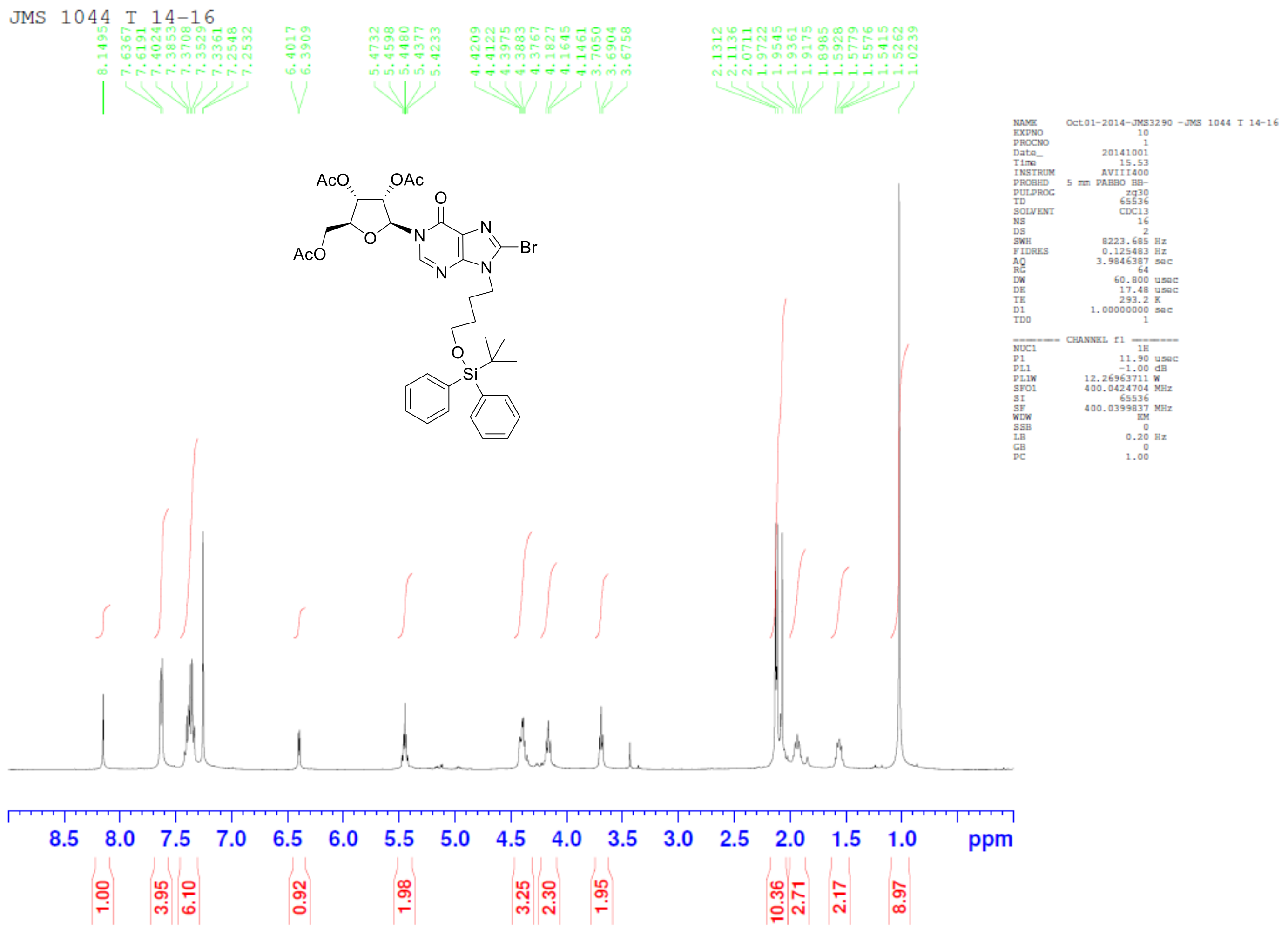
JMS 1044 T 14-16
8.1495
7.6367
7.6191
7.4024
7.3853
7.3708
7.3529
7.3361
7.2548
7.2532
6.4017
6.3909
5.4732
5.4598
5.4480
5.4377
5.4233
4.4209
4.4122
4.3975
4.3883
4.3767
4.1827
4.1645
4.1461
3.7050
3.6904
3.6758
2.1312
2.1136
2.0711
1.9722
1.9545
1.9361
1.9175
1.8985
1.5928
1.5779
1.5576
1.5415
1.5262
1.0239
AcO
OAc
O
N
N
Br
AcO
O
N
N
O
Si
NAME Oct01-2014-JMS3290 -JMS 1044 T 14-16
EXPNO 10
PROCNO 1
Date_ 20141001
Time 15.53
INSTRUM AVIII400
PROBHD 5 mm PABBO BB-
PULPROG zg30
TD 65536
SOLVENT CDCl3
NS 16
DS 2
SWH 8223.685 Hz
FIDRES 0.125483 Hz
AQ 3.9846387 sec
RG 64
DW 60.800 usec
DE 17.48 usec
TE 293.2 K
D1 1.00000000 sec
TD0 1
======== CHANNEL f1 ========
NUC1 1H
P1 11.90 usec
PL1 -1.00 dB
PL1W 12.26963711 W
SFO1 400.0424704 MHz
SI 65536
SF 400.0399837 MHz
WDW EM
SSB 0
LB 0.20 Hz
GB 0
PC 1.00
8.5 8.0 7.5 7.0 6.5 6.0 5.5 5.0 4.5 4.0 3.5 3.0 2.5 2.0 1.5 1.0 ppm
1.00
3.95
6.10
0.92
1.98
3.25
2.30
1.95
10.36
2.71
2.17
8.97

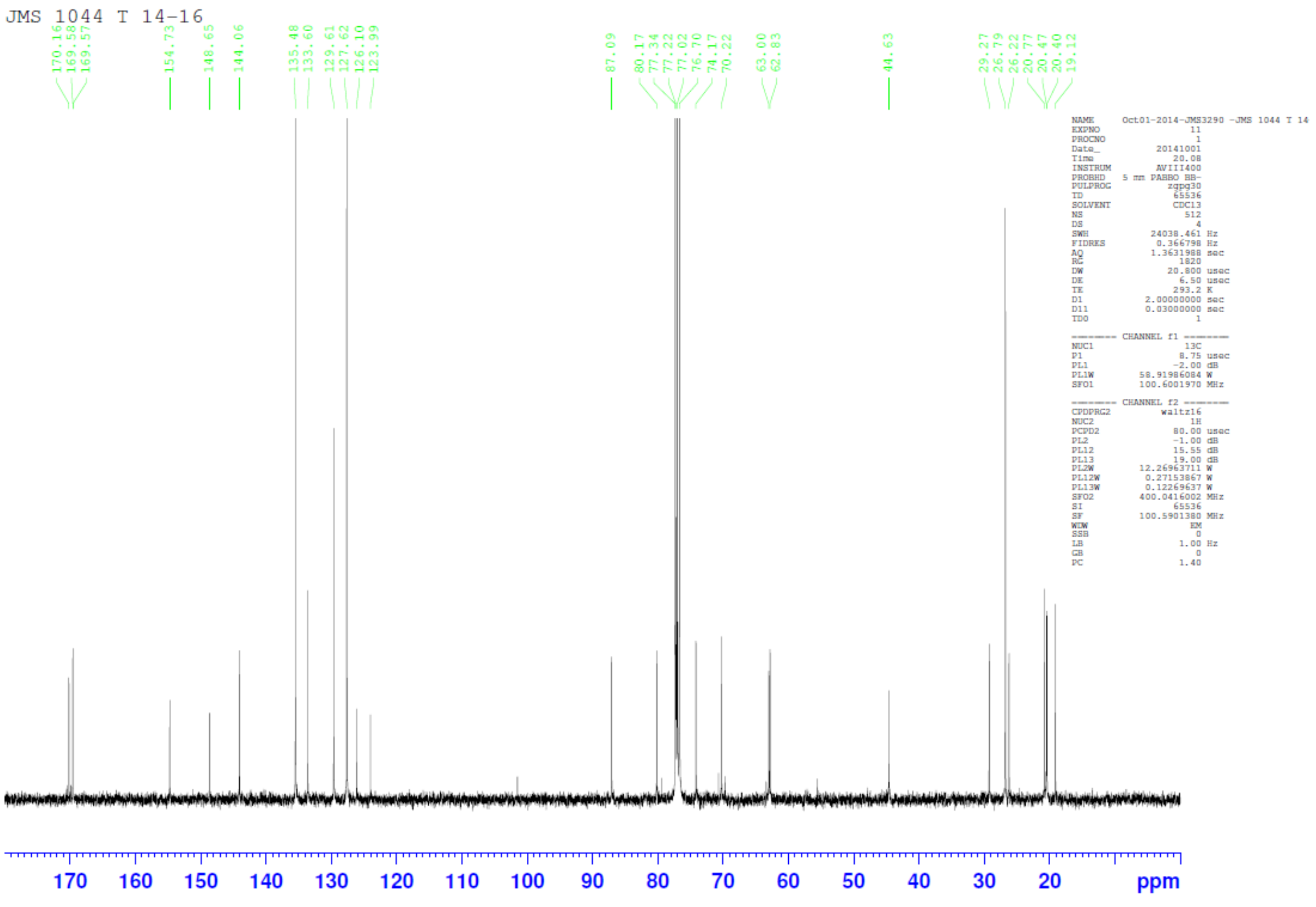
JMS 1044 T 14-16
170.16
169.58
169.57
154.73
148.65
144.06
135.48
133.60
129.61
127.62
126.10
123.99
87.09
80.17
77.34
77.22
77.02
76.70
74.17
70.22
63.00
62.83
44.63
29.27
26.79
26.22
20.77
20.47
20.40
19.12
NAME Oct01-2014-JMS3290 -JMS 1044 T 14
EXPNO 11
PROCNO 1
Date_ 20141001
Time 20.08
INSTRUM AVIII400
PROBHD 5 mm PABBO BB-
PULPROG zgpg30
TD 65536
SOLVENT CDCl3
NS 512
DS 4
SWH 24038.461 Hz
FIDRES 0.366798 Hz
AQ 1.3631988 sec
RG 1820
DW 20.800 usec
DE 6.50 usec
TE 293.2 K
D1 2.00000000 sec
D11 0.03000000 sec
TD0 1
======== CHANNEL f1 ========
NUC1 13C
P1 8.75 usec
PL1 -2.00 dB
PL1W 58.91986084 W
SFO1 100.6001970 MHz
======== CHANNEL f2 ========
CPDPRG2 waltz16
NUC2 1H
PCPD2 80.00 usec
PL2 -1.00 dB
PL12 15.55 dB
PL13 19.00 dB
PL2W 12.26963711 W
PL12W 0.27153867 W
PL13W 0.12269637 W
SFO2 400.0416002 MHz
SI 65536
SF 100.5901380 MHz
WDW EM
SSB 0
LB 1.00 Hz
GB 0
PC 1.40
170 160 150 140 130 120 110 100 90 80 70 60 50 40 30 20 ppm

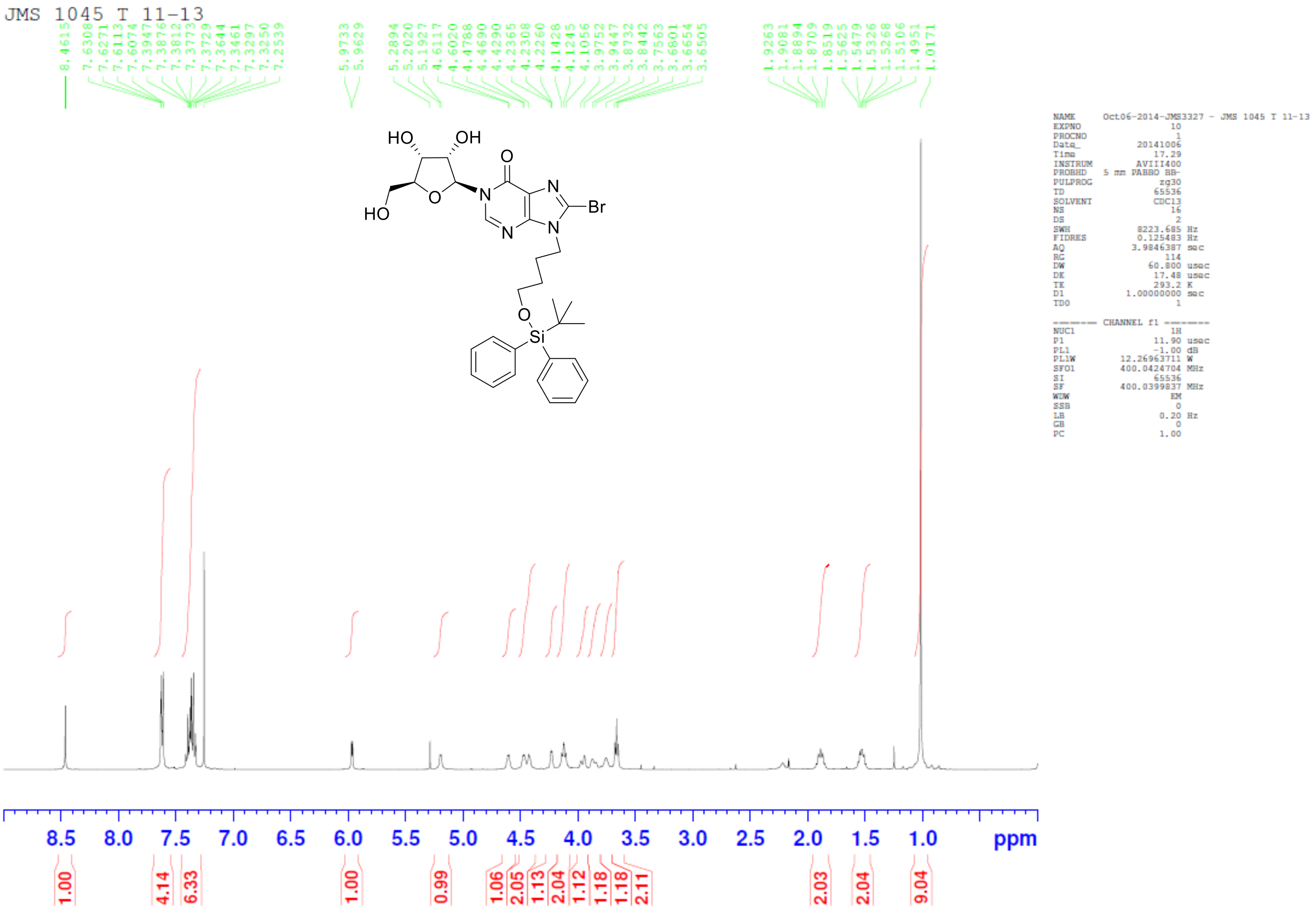
JMS 1045 T 11-13
8.4615
7.6308
7.6271
7.6113
7.6074
7.3947
7.3876
7.3812
7.3773
7.3729
7.3644
7.3461
7.3297
7.3250
7.2539
5.9733
5.9629
5.2894
5.2020
5.1927
4.6117
4.6020
4.4788
4.4690
4.4290
4.2365
4.2308
4.2260
4.1428
4.1245
4.1056
3.9752
3.9447
3.8732
3.8442
3.7563
3.6801
3.6654
3.6505
1.9263
1.9081
1.8894
1.8709
1.8519
1.5625
1.5479
1.5326
1.5268
1.5106
1.4951
1.0171
HO
OH
O
HO
O
N
N
N
N
Br
O
Si
NAME Oct06-2014-JMS3327 - JMS 1045 T 11-13
EXPNO 10
PROCNO 1
Date_ 20141006
Time 17.29
INSTRUM AVIII400
PROBHD 5 mm PABBO BB-
PULPROG zg30
TD 65536
SOLVENT CDCl3
NS 16
DS 2
SWH 8223.685 Hz
FIDRES 0.125483 Hz
AQ 3.9846387 sec
RG 114
DW 60.800 usec
DE 17.48 usec
TE 293.2 K
D1 1.00000000 sec
TD0 1
CHANNEL f1
NUC1 1H
P1 11.90 usec
PL1 -1.00 dB
PL1W 12.26963711 W
SFO1 400.0424704 MHz
SI 65536
SF 400.0399837 MHz
WDW EM
SSB 0
LB 0.20 Hz
GB 0
PC 1.00
8.5
8.0
7.5
7.0
6.5
6.0
5.5
5.0
4.5
4.0
3.5
3.0
2.5
2.0
1.5
1.0
ppm
1.00
4.14
6.33
1.00
0.99
1.06
2.05
1.13
2.04
1.12
1.18
1.18
2.11
2.03
2.04
9.04

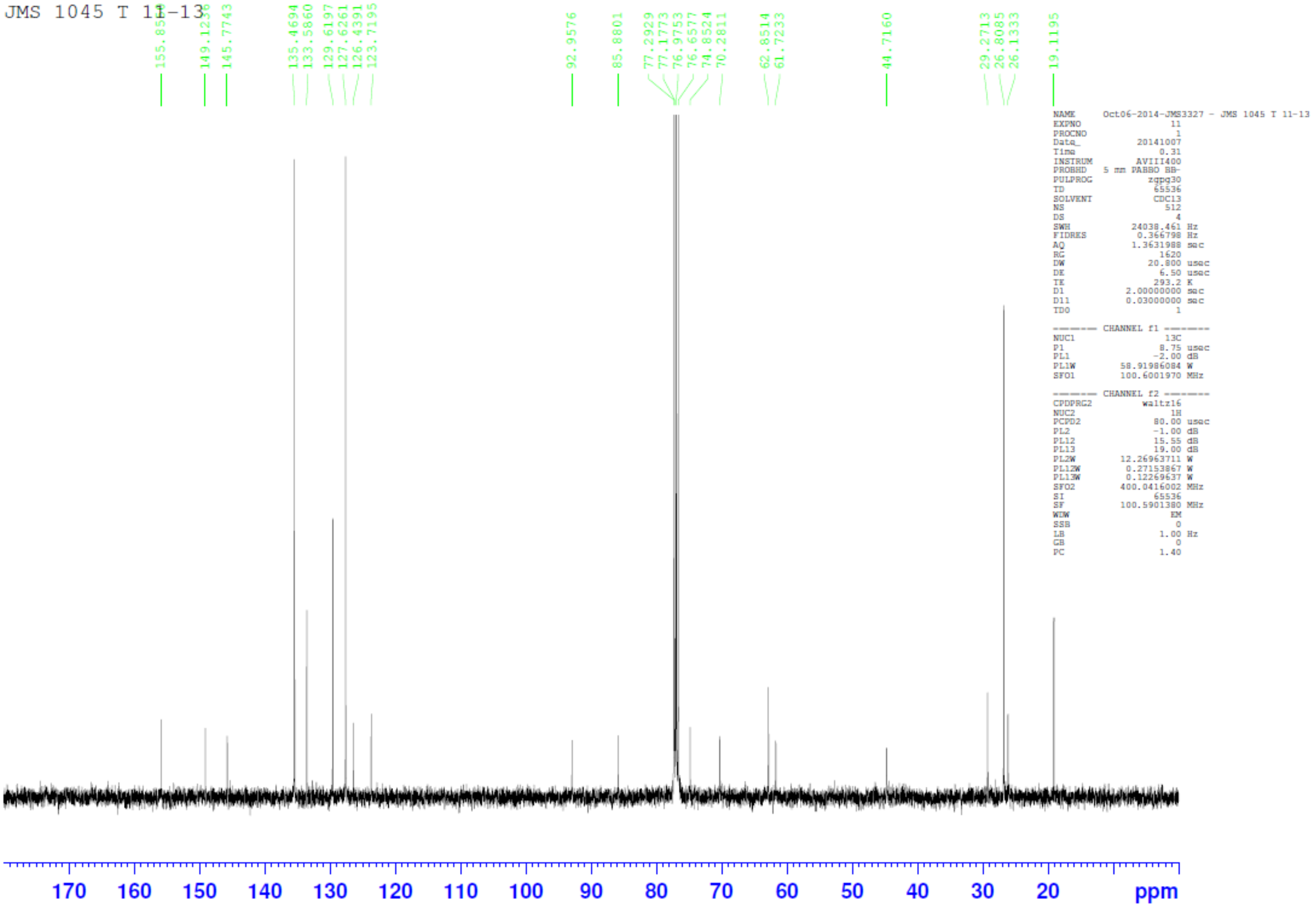
JMS 1045 T 11-13
155.85
149.12
145.7743
135.4694
133.5860
129.6197
127.6261
126.4391
123.7195
92.9576
85.8801
77.2929
77.1773
76.9753
76.6577
74.8524
70.2811
62.8514
61.7233
44.7160
29.2713
26.8085
26.1333
19.1195
NAME Oct06-2014-JMS3327 - JMS 1045 T 11-13
EXPNO 11
PROCNO 1
Date_ 20141007
Time 0.31
INSTRUM AVIII400
PROBHD 5 mm PABBO BB-
PULPROG zgpg30
TD 65536
SOLVENT CDCl3
NS 512
DS 4
SWH 24038.461 Hz
FIDRES 0.366798 Hz
AQ 1.3631988 sec
RG 1620
DW 20.800 usec
DE 6.50 usec
TE 293.2 K
D1 2.00000000 sec
D11 0.03000000 sec
TD0 1
CHANNEL f1
NUC1 13C
P1 8.75 usec
PL1 -2.00 dB
PL1W 58.91986084 W
SFO1 100.6001970 MHz
CHANNEL f2
CPDPRG2 waltz16
NUC2 1H
PCPD2 80.00 usec
PL2 -1.00 dB
PL12 15.55 dB
PL13 19.00 dB
PL2W 12.26963711 W
PL12W 0.27153867 W
PL13W 0.12269637 W
SFO2 400.0416002 MHz
SI 65536
SF 100.5901380 MHz
WDW EM
SSB 0
LB 1.00 Hz
GB 0
PC 1.40
170
160
150
140
130
120
110
100
90
80
70
60
50
40
30
20
ppm

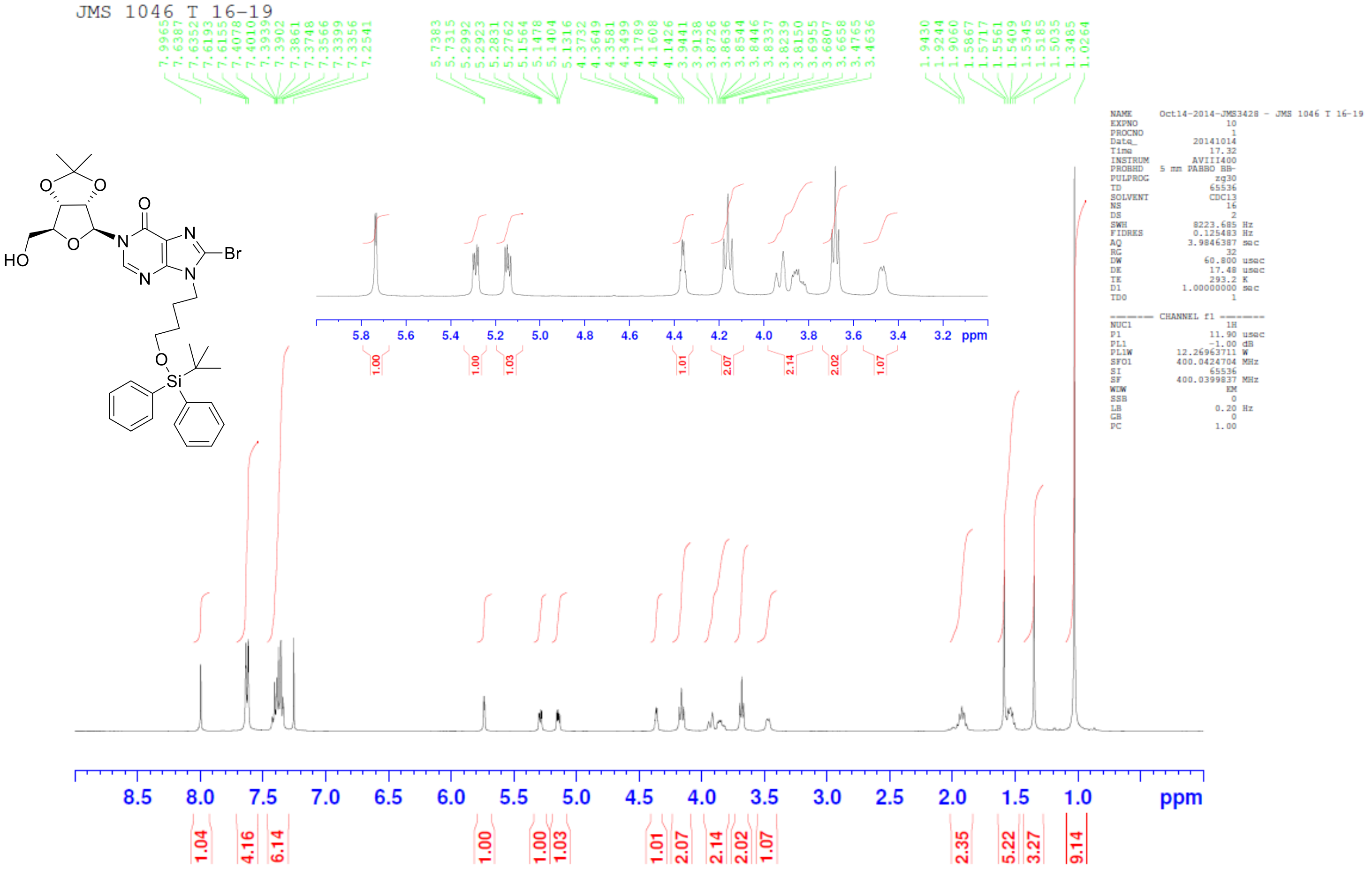

JMS 1046 T 16-19
7.9965 7.6387 7.6352 7.6193 7.6155 7.4078 7.4010 7.3939 7.3902 7.3861 7.3748 7.3566 7.3399 7.3356 7.2541
5.7383 5.7315 5.2992 5.2923 5.2831 5.2762 5.1564 5.1478 5.1404 5.1316 4.3732 4.3649 4.3581 4.3499 4.1789 4.1608 4.1426 3.9441 3.9138 3.8726 3.8636 3.8544 3.8446 3.8337 3.8239 3.8150 3.6955 3.6807 3.6658 3.4765 3.4636
1.9430 1.9244 1.9060 1.5867 1.5717 1.5561 1.5409 1.5345 1.5185 1.5035 1.3485 1.0264
NAME Oct14-2014-JMS3428 - JMS 1046 T 16-19
EXPNO 10
PROCNO 1
Date_ 20141014
Time 17.32
INSTRUM AVIII400
PROBHD 5 mm PABBO BB-
PULPROG zg30
TD 65536
SOLVENT CDCl3
NS 16
DS 2
SWH 8223.685 Hz
FIDRES 0.125483 Hz
AQ 3.9846387 sec
RG 32
DW 60.800 usec
DE 17.48 usec
TE 293.2 K
D1 1.00000000 sec
TD0 1
======== CHANNEL f1 ========
NUC1 1H
P1 11.90 usec
PL1 -1.00 dB
PL1W 12.26963711 W
SFO1 400.0424704 MHz
SI 65536
SF 400.0399837 MHz
WDW EM
SSB 0
LB 0.20 Hz
GB 0
PC 1.00
5.8 5.6 5.4 5.2 5.0 4.8 4.6 4.4 4.2 4.0 3.8 3.6 3.4 3.2 ppm
1.00 1.00 1.03 1.01 2.07 2.14 2.02 1.07
8.5 8.0 7.5 7.0 6.5 6.0 5.5 5.0 4.5 4.0 3.5 3.0 2.5 2.0 1.5 1.0 ppm
1.04 4.16 6.14 1.00 1.00 1.03 1.01 2.07 2.14 2.02 1.07 2.35 5.22 3.27 9.14

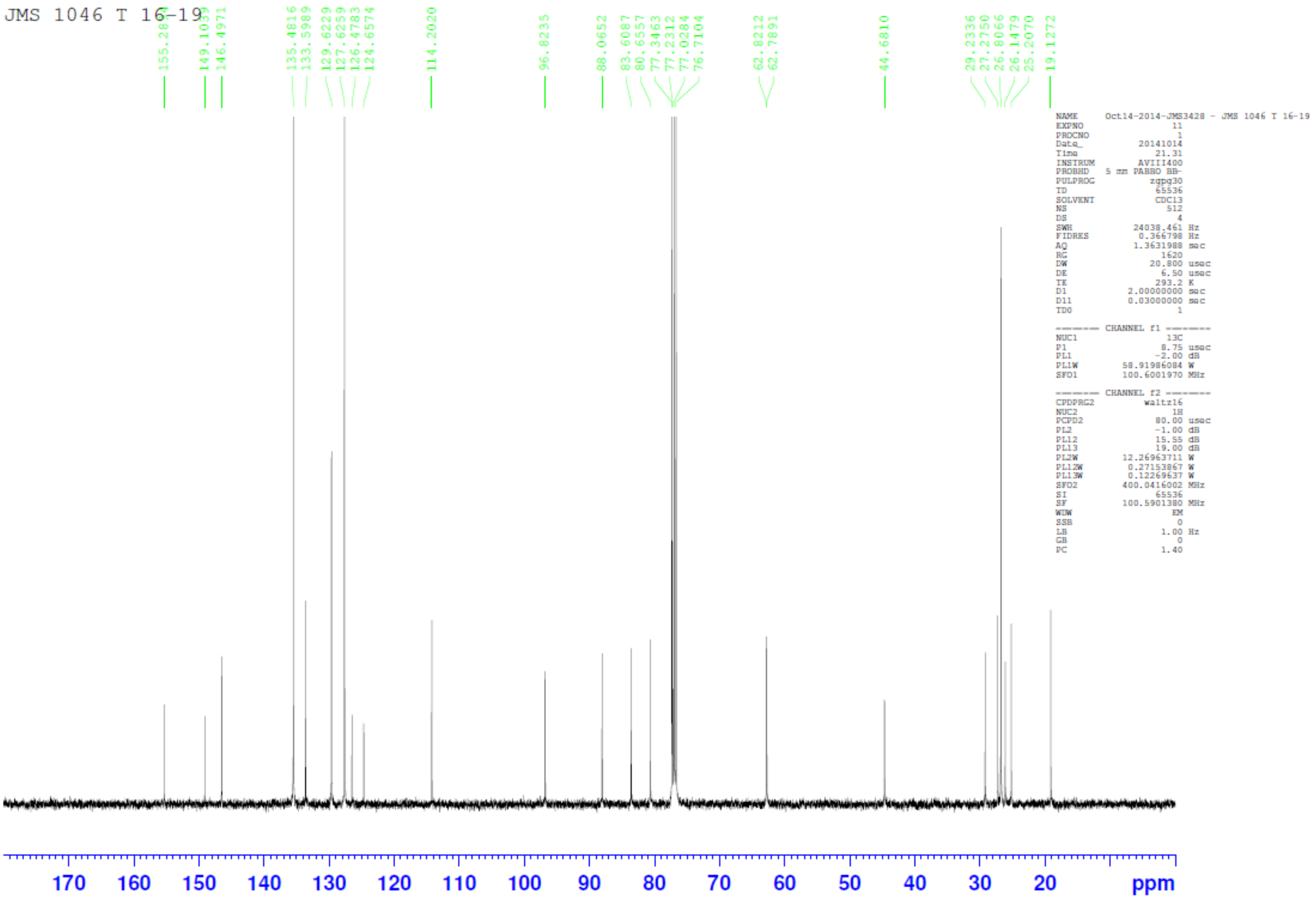

JMS 1046 T 16-19
149.1039 146.4971 135.4816 133.5989 129.6229 127.6259 126.4783 124.6574 114.2020 96.8235 88.0652 83.6087 80.6557 77.3463 77.2312 77.0284 76.7104 62.8212 62.7891 44.6810 29.2336 27.2750 26.8666 26.1479 25.2070 19.1272
NAME Oct14-2014-JMS3428 - JMS 1046 T 16-19
EXPNO 11
PROCNO 1
Date_ 20141014
Time 21.31
INSTRUM AVIII400
PROBHD 5 mm PABBO BB-
PULPROG zgpg30
TD 65536
SOLVENT CDCl3
NS 512
DS 4
SWH 24038.461 Hz
FIDRES 0.366798 Hz
AQ 1.3631988 sec
RG 1620
DW 20.800 usec
DE 6.50 usec
TE 293.2 K
D1 2.00000000 sec
D11 0.03000000 sec
TD0 1
======== CHANNEL f1 ========
NUC1 13C
P1 8.75 usec
PL1 -2.00 dB
PL1W 58.91986084 W
SFO1 100.6001970 MHz
======== CHANNEL f2 ========
CPDPRG2 waltz16
NUC2 1H
PCPD2 80.00 usec
PL2 -1.00 dB
PL12 15.55 dB
PL13 19.00 dB
PL2W 12.26963711 W
PL12W 0.27153867 W
PL13W 0.12269637 W
SFO2 400.0416002 MHz
SI 65536
SF 100.5901380 MHz
WDW EM
SSB 0
LB 1.00 Hz
GB 0
PC 1.40
170 160 150 140 130 120 110 100 90 80 70 60 50 40 30 20 ppm

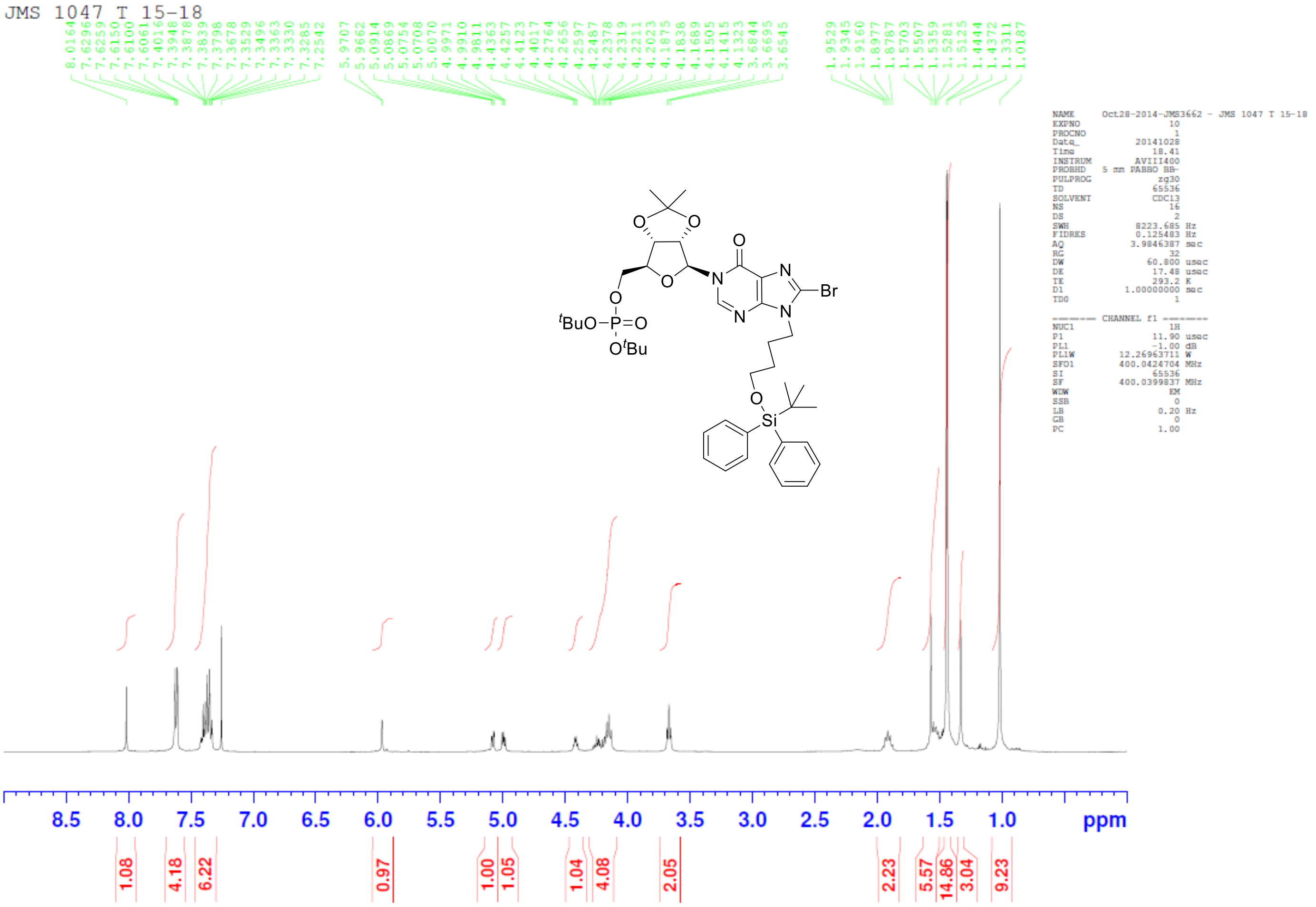
JMS 1047 T 15-18
8.0164
7.6296
7.6259
7.6150
7.6100
7.6061
7.4016
7.3948
7.3878
7.3839
7.3798
7.3678
7.3529
7.3496
7.3363
7.3330
7.3285
7.2542
5.9707
5.9662
5.0914
5.0869
5.0754
5.0708
5.0070
4.9971
4.9910
4.9811
4.4363
4.4257
4.4123
4.4017
4.2764
4.2656
4.2597
4.2487
4.2378
4.2319
4.2211
4.2023
4.1875
4.1838
4.1689
4.1505
4.1415
4.1323
3.6844
3.6695
3.6545
1.9529
1.9345
1.9160
1.8977
1.8787
1.5703
1.5507
1.5359
1.5281
1.5125
1.4444
1.4372
1.3311
1.0187
O
N
N
Br
N
N
O
O
O
O
tBuO–P=O
OtBu
O
Si
8.5
8.0
7.5
7.0
6.5
6.0
5.5
5.0
4.5
4.0
3.5
3.0
2.5
2.0
1.5
1.0
ppm
1.08
4.18
6.22
0.97
1.00
1.05
1.04
4.08
2.05
2.23
5.57
14.86
3.04
9.23

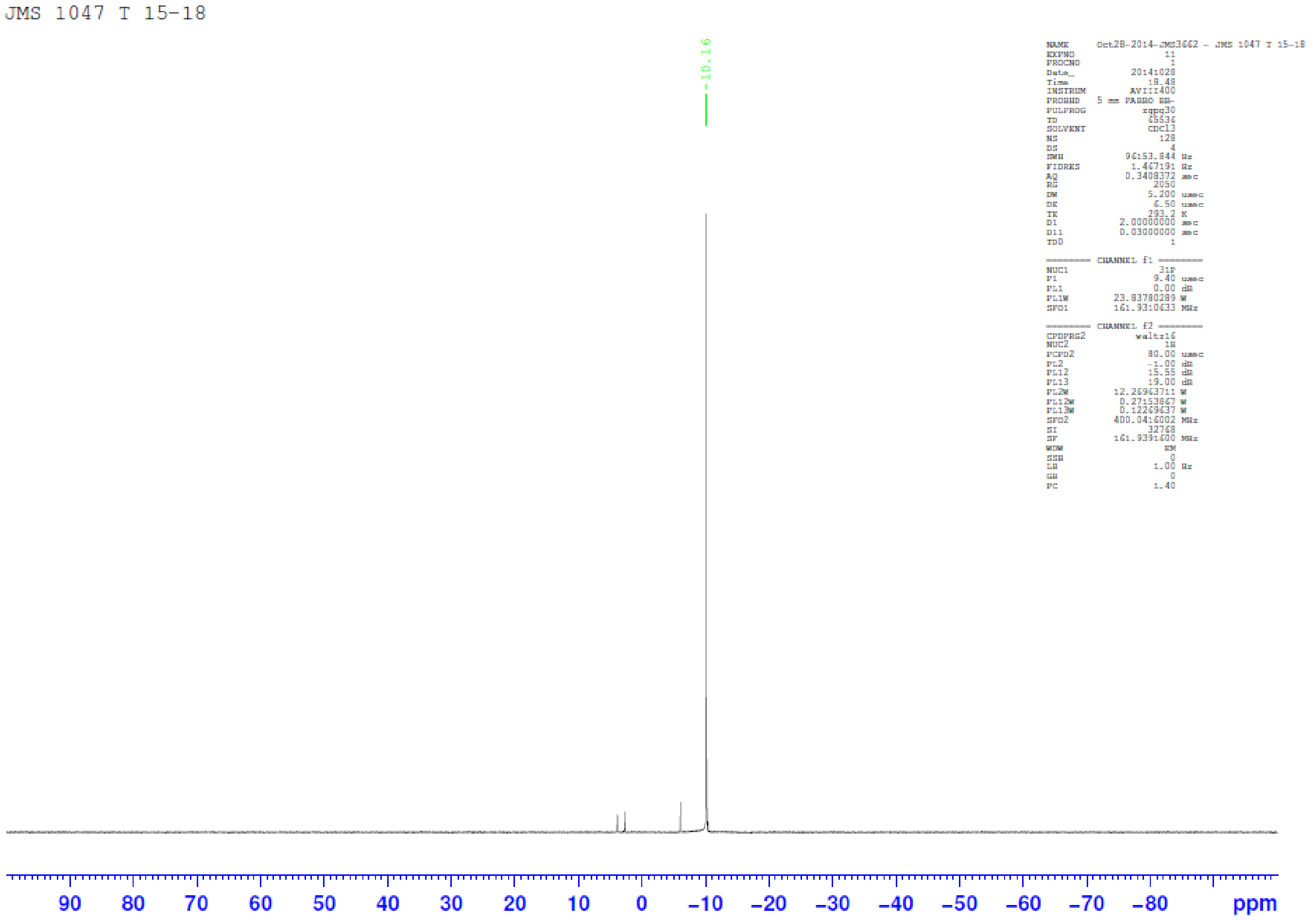
JMS 1047 T 15-18
-10.16
90
80
70
60
50
40
30
20
10
0
-10
-20
-30
-40
-50
-60
-70
-80
ppm

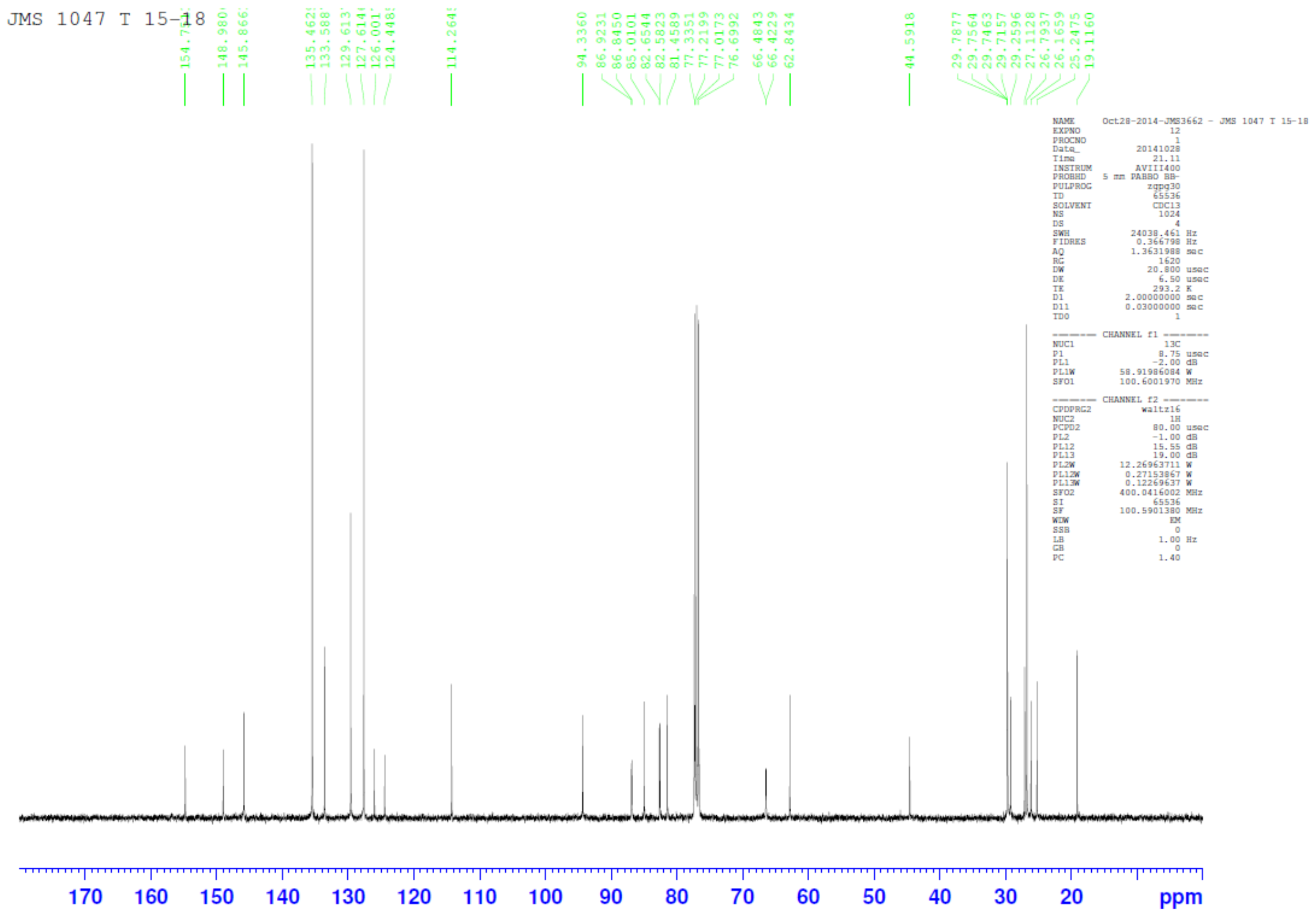
JMS 1047 T 15-18
ppm

JMS1051 T17-19

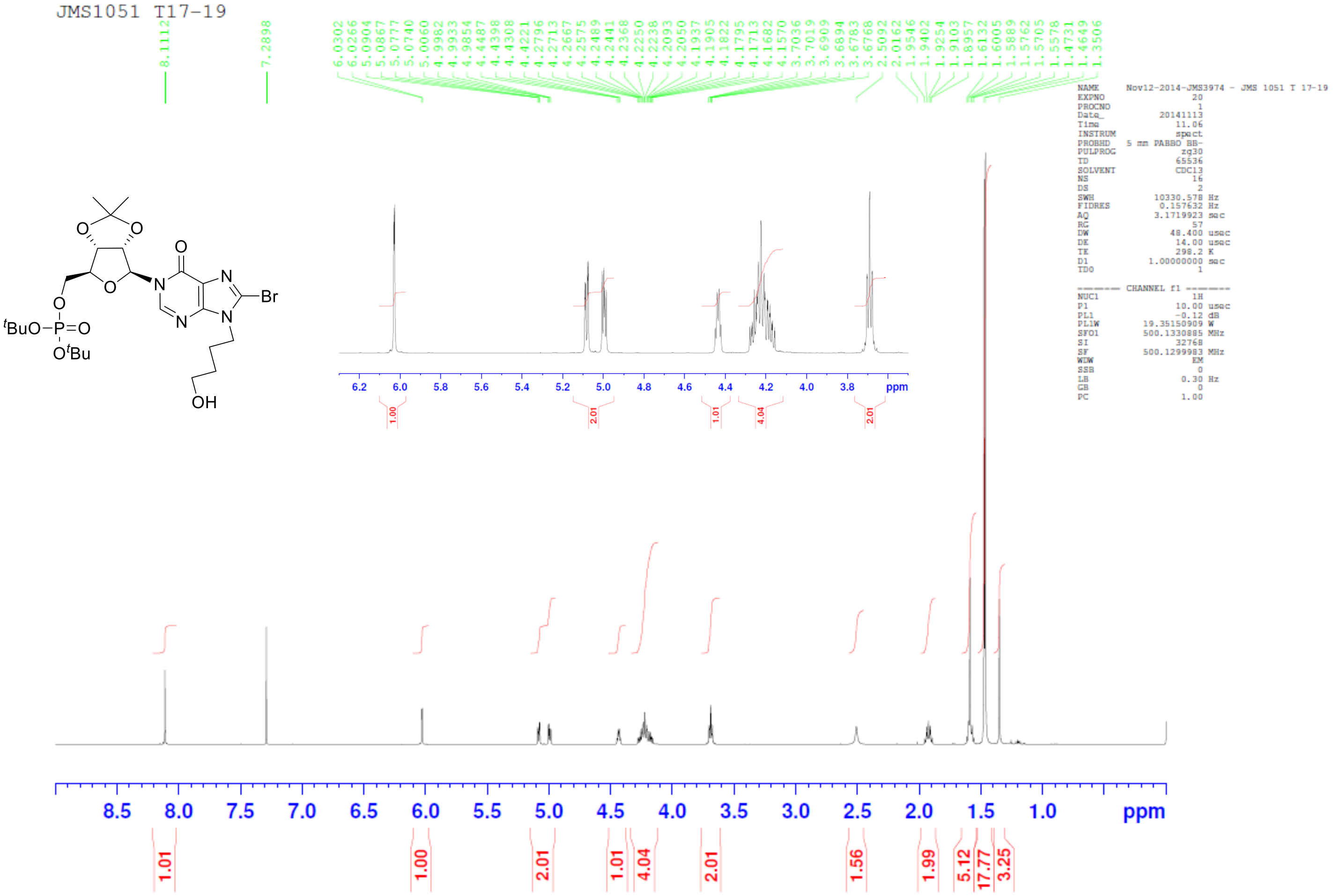

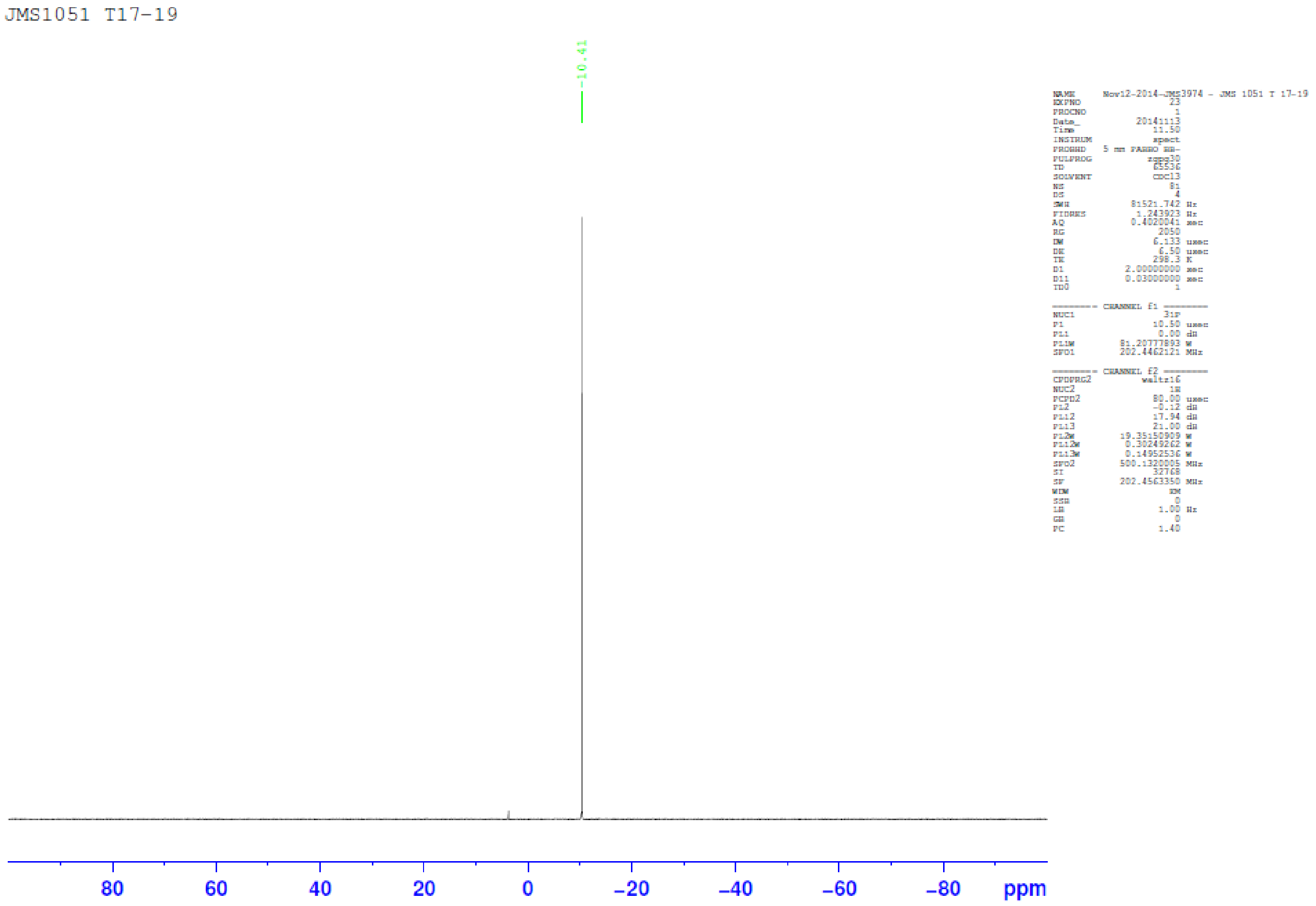

JMS1051 T17-19
-10.41
80
60
40
20
0
-20
-40
-60
-80
ppm

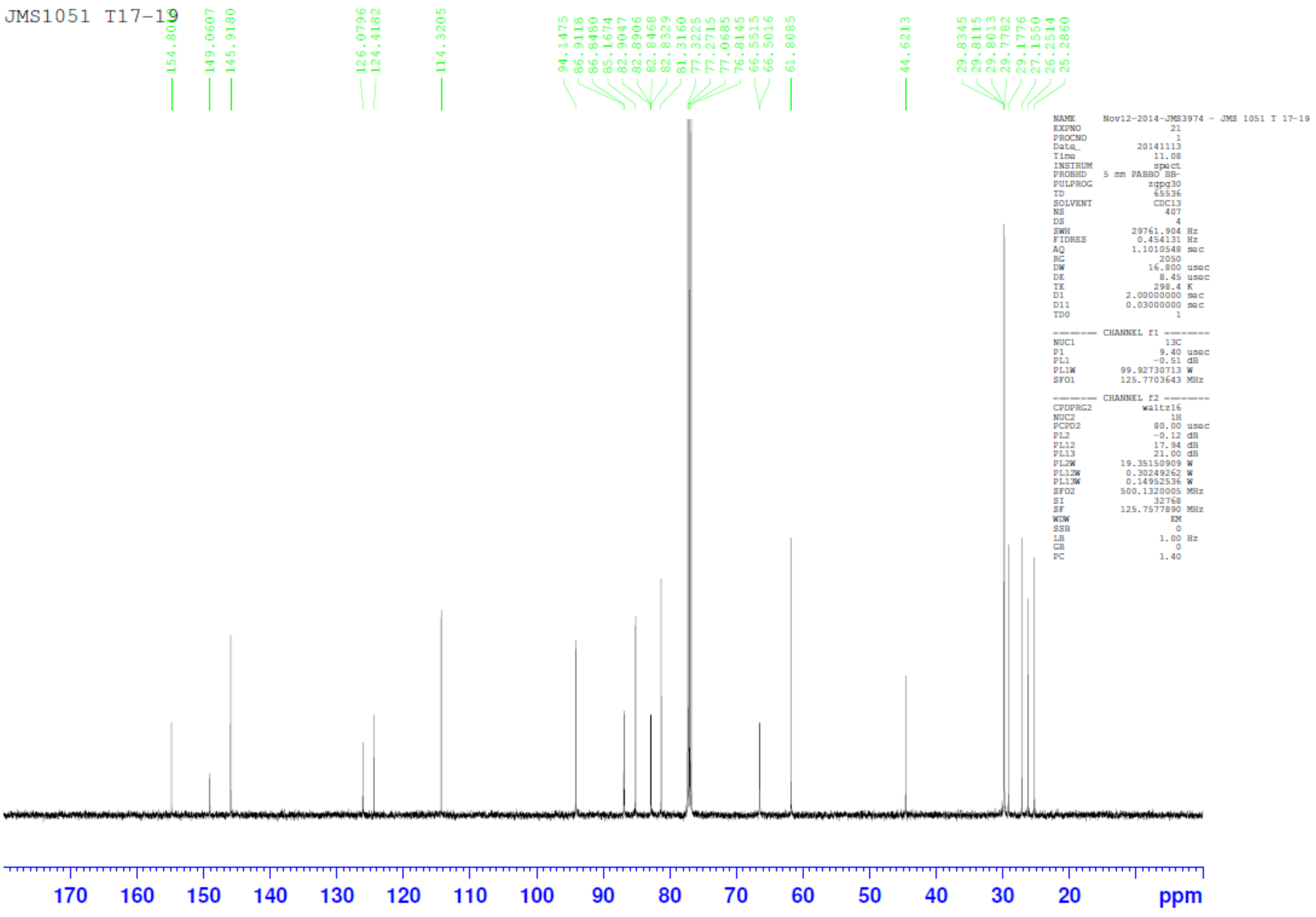

JMS1051 T17-19
154.8009
149.0607
145.9180
126.0796
124.4182
114.3205
94.1475
86.9118
86.8480
85.1674
82.9047
82.8906
82.8468
82.8329
81.3160
77.3225
77.2715
77.0685
76.8145
66.5515
66.5016
61.8085
44.6213
29.8345
29.8115
29.8013
29.7782
29.1776
27.1550
26.2514
25.2860
170
160
150
140
130
120
110
100
90
80
70
60
50
40
30
20
ppm

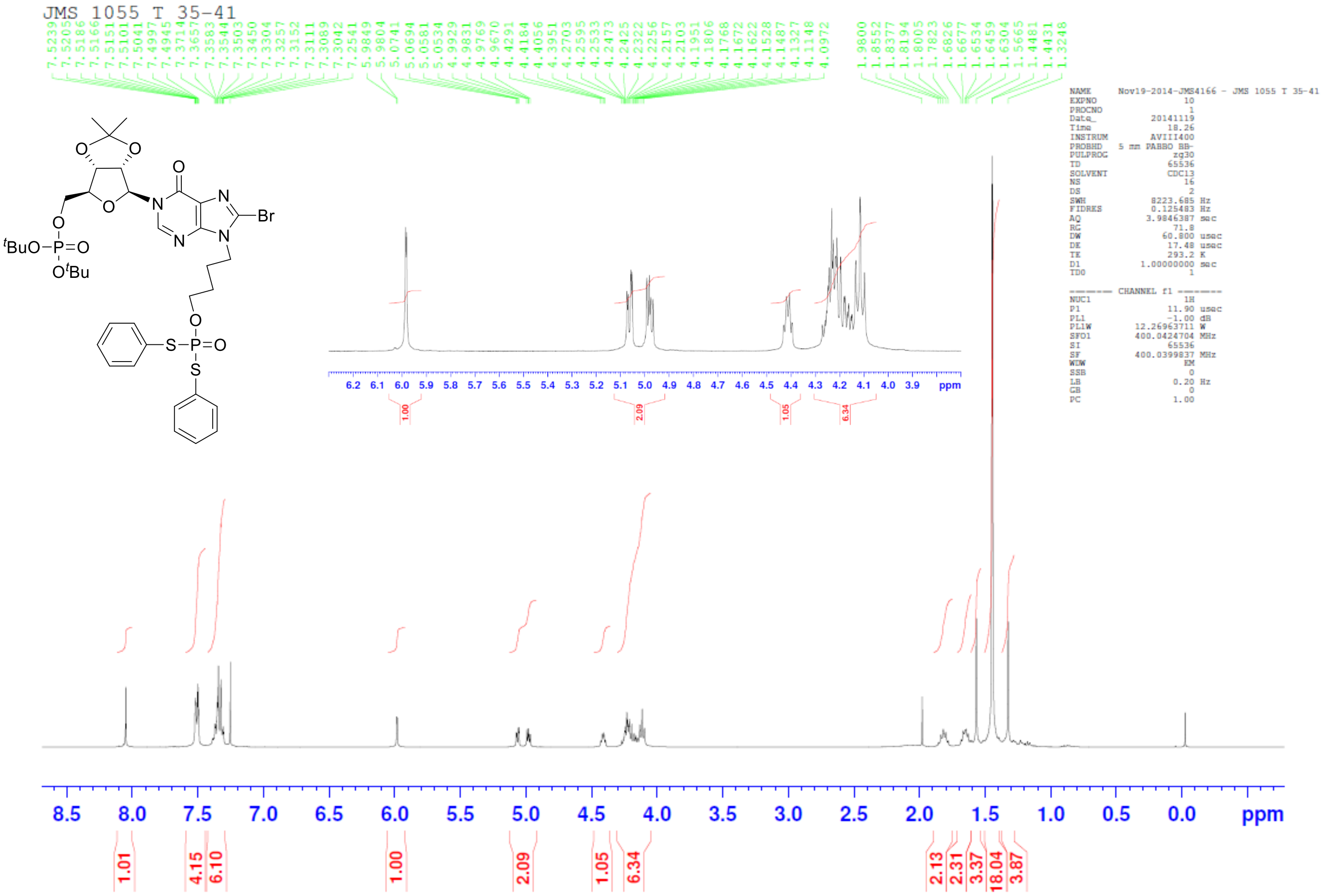
JMS 1055 T 35-41
7.5239 7.5205 7.5186 7.5166 7.5151 7.5101 7.5041 7.4997 7.4945 7.3714 7.3657 7.3583 7.3544 7.3503 7.3450 7.3304 7.3257 7.3152 7.3111 7.3089 7.3042 7.2541 5.9849 5.9804 5.0741 5.0694 5.0581 5.0534 4.9929 4.9831 4.9769 4.9670 4.4291 4.4184 4.4056 4.3951 4.2703 4.2595 4.2533 4.2473 4.2425 4.2322 4.2256 4.2157 4.2103 4.1951 4.1806 4.1768 4.1672 4.1622 4.1528 4.1487 4.1327 4.1148 4.0972
1.9600 1.8552 1.8377 1.8194 1.8065 1.7823 1.6826 1.6677 1.6534 1.6459 1.6304 1.5665 1.4481 1.4431 1.3248
Br
1.01 4.15 6.10 1.00 2.09 1.05 6.34 2.13 2.31 3.37 18.04 3.87
ppm

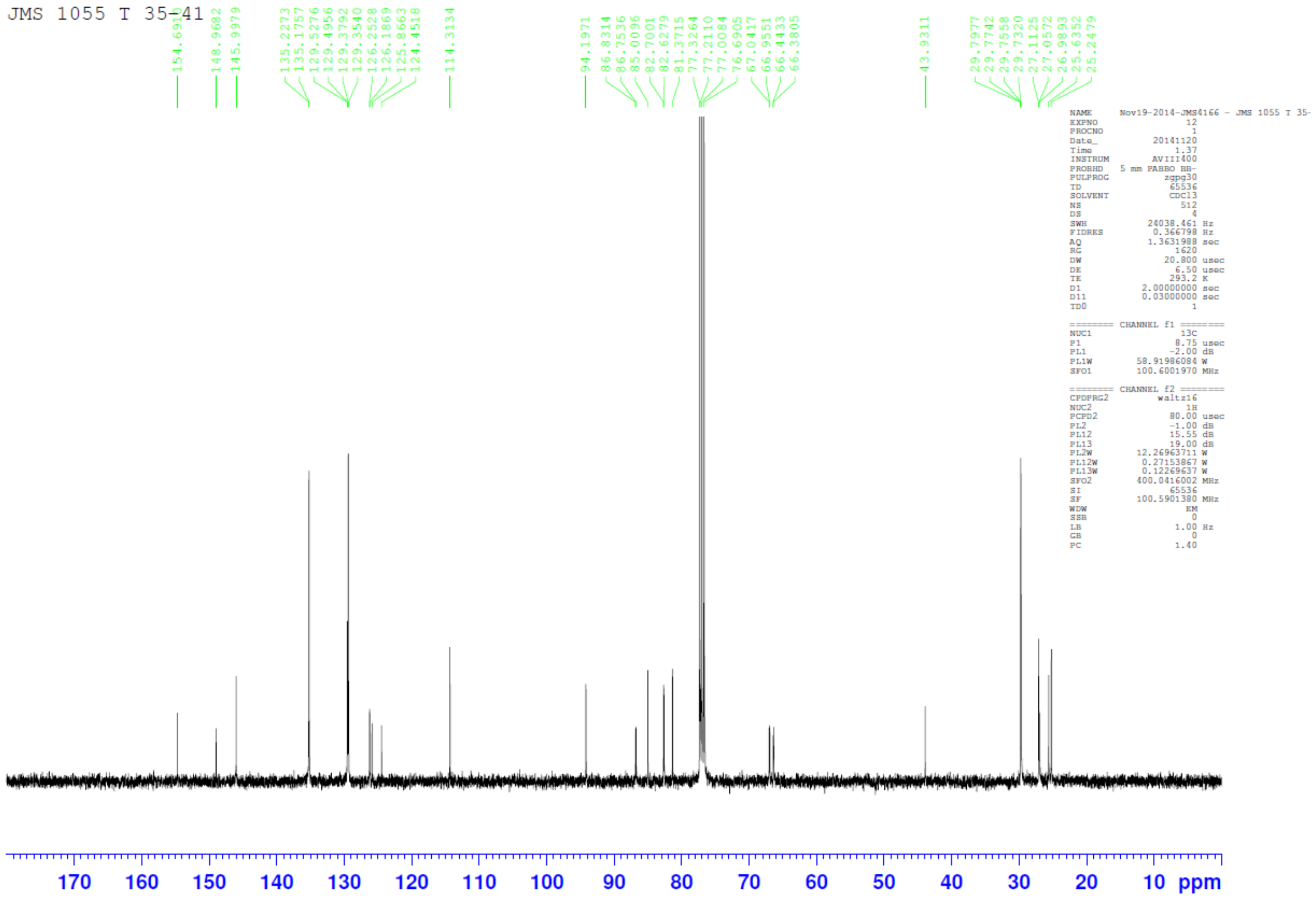
JMS 1055 T 35-41
154.6911 148.9682 145.9979 135.2273 135.1757 129.5276 129.4956 129.3792 129.3540 126.2528 126.1869 125.8663 124.4518 114.3134 94.1971 86.8314 86.7536 85.0096 82.7001 82.6279 81.3715 77.3264 77.2110 77.0084 76.6905 67.0417 66.9551 66.4433 66.3805 43.9311 29.7977 29.7742 29.7558 29.7320 27.1125 27.0572 26.9893 25.6352 25.2479
ppm

JMS 1055 T 35-41

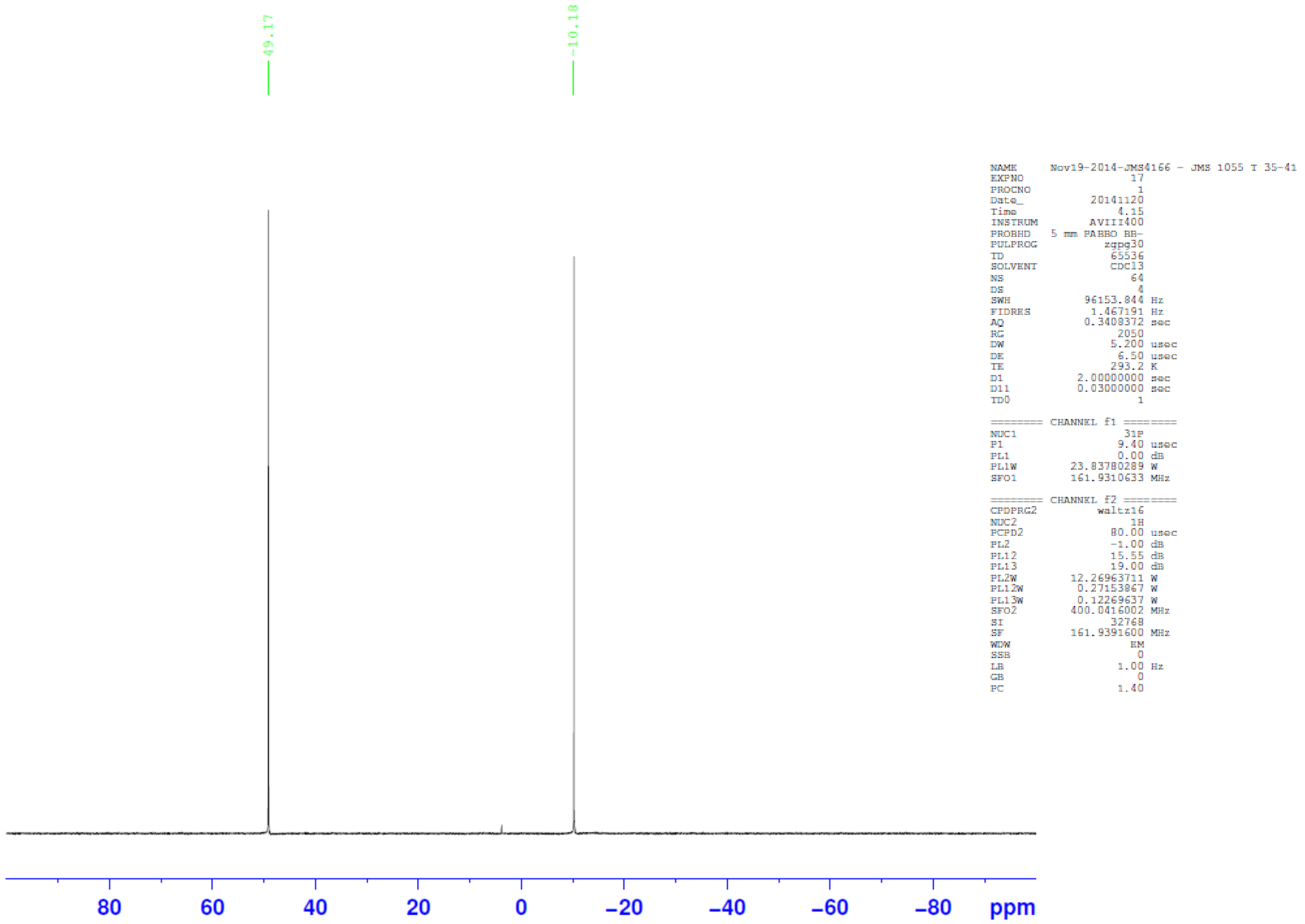

JMS 1059

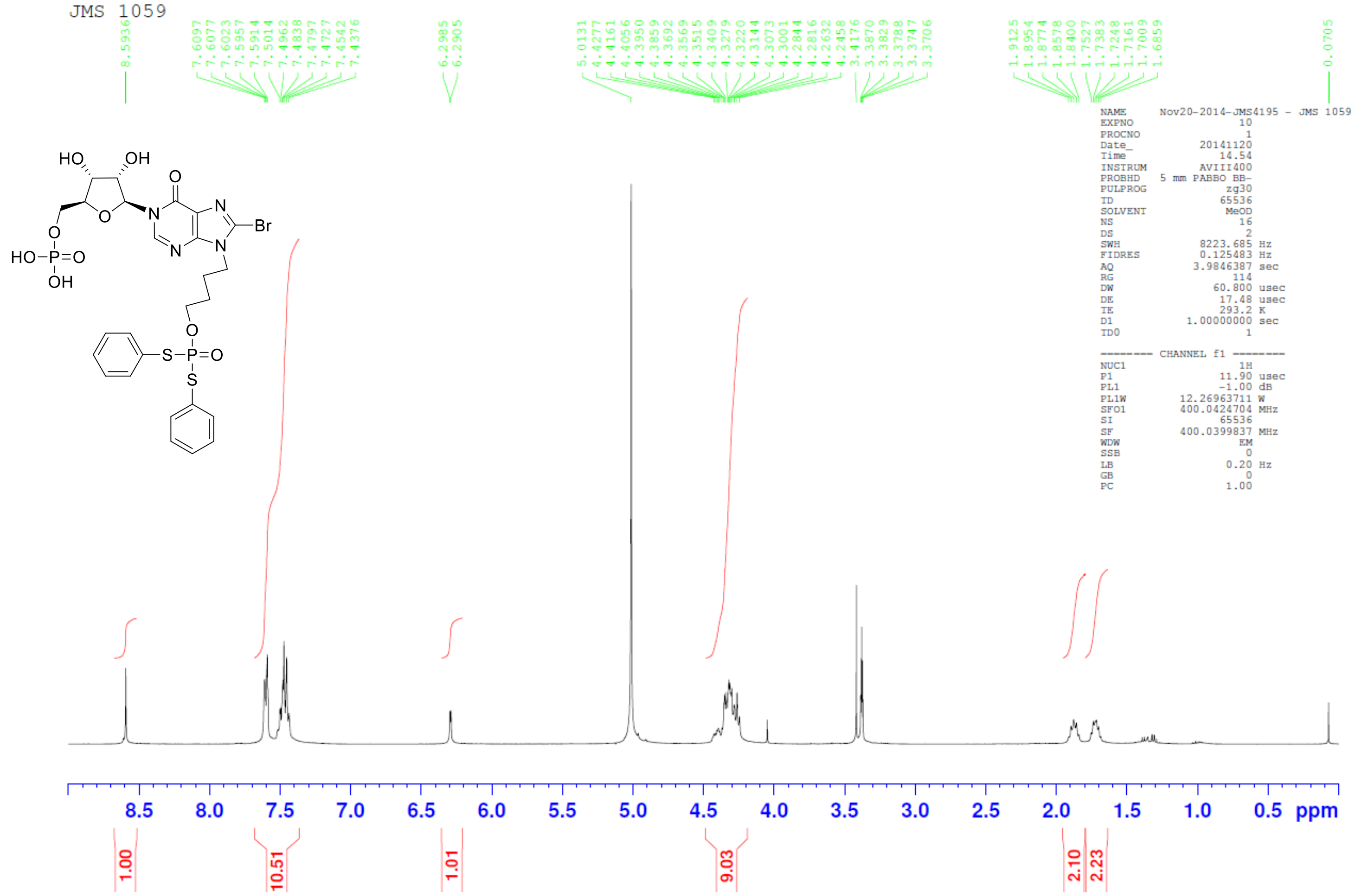

JMS 1059

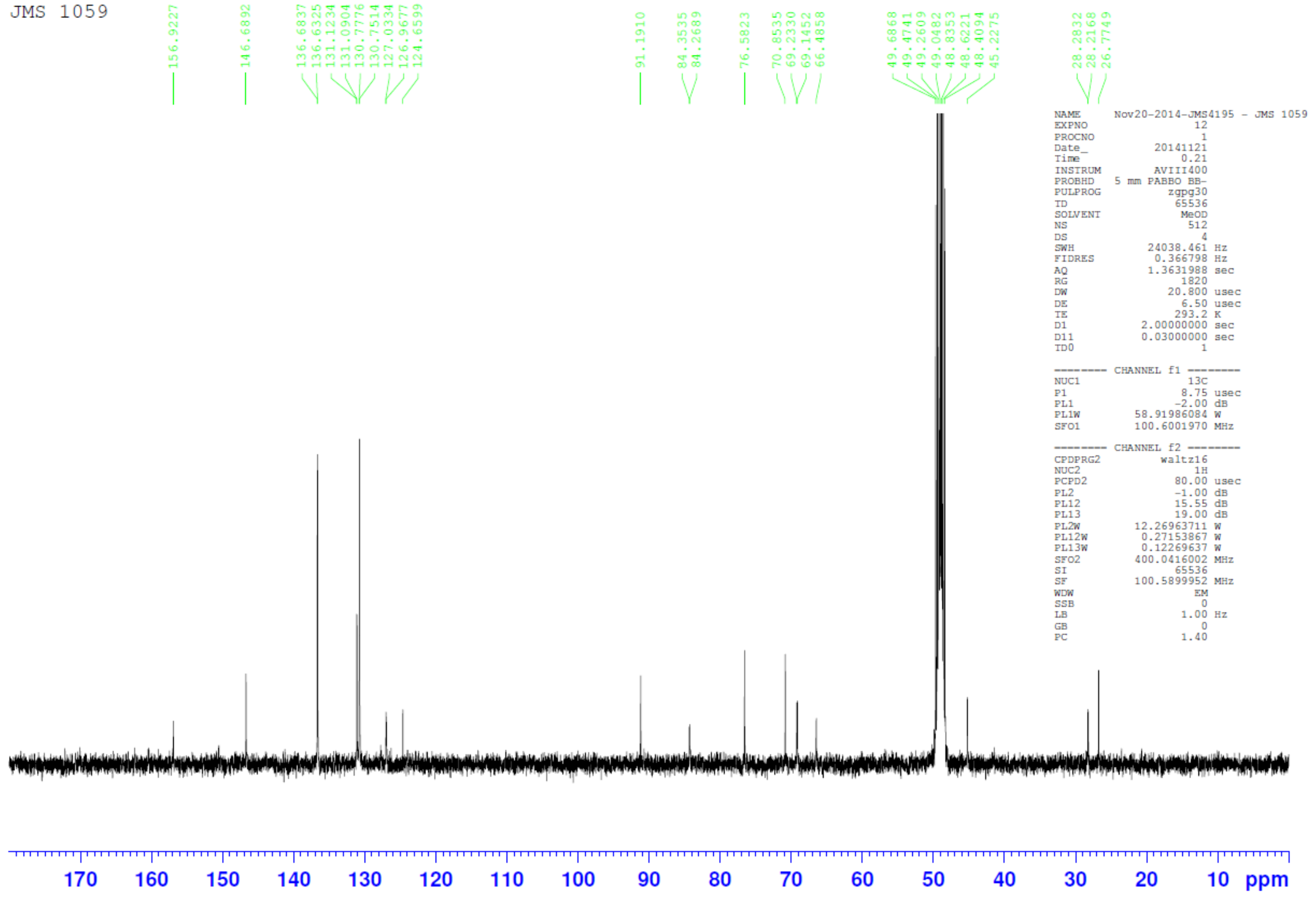

JMS 1059

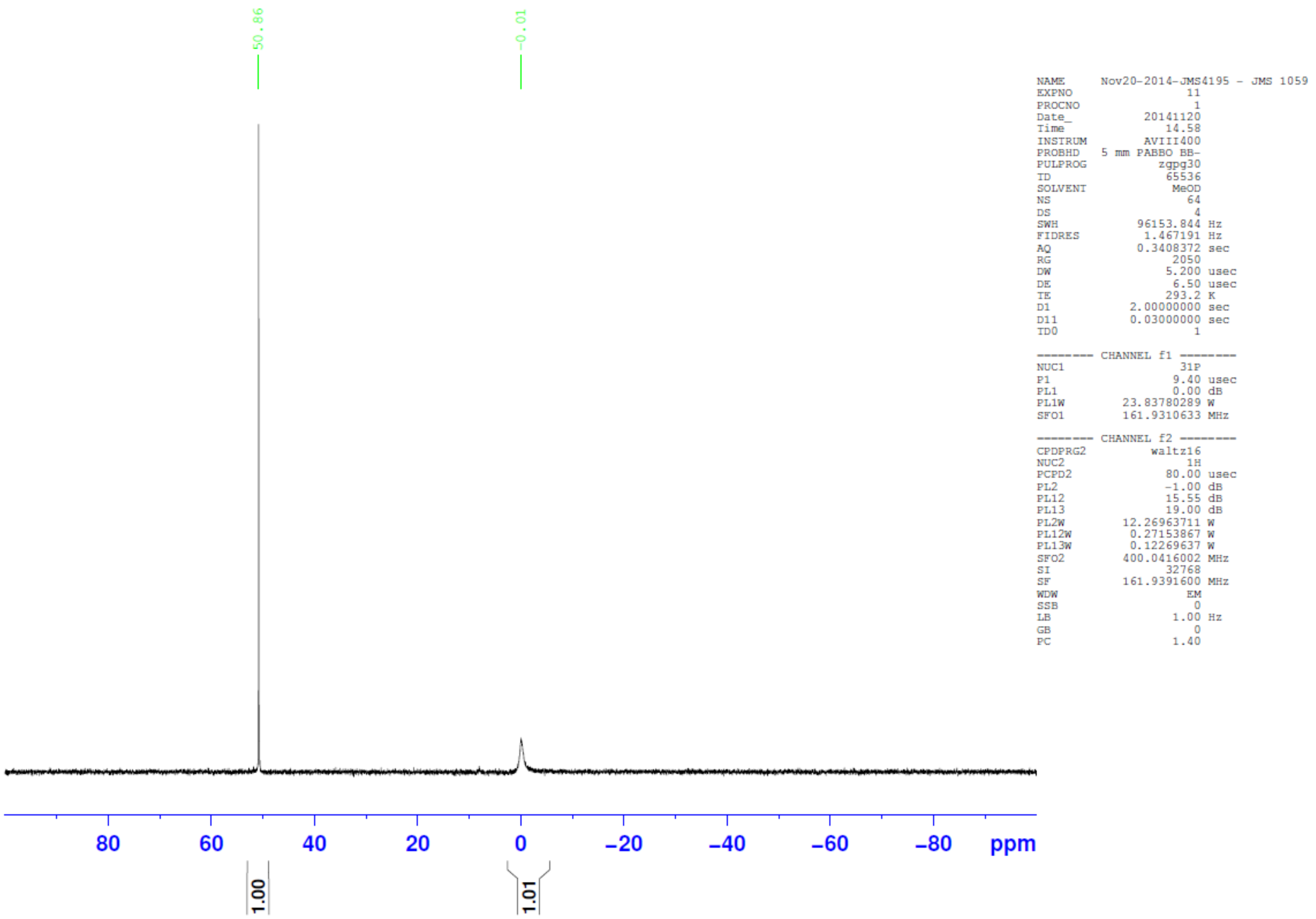
50.86
-0.01
NAME Nov20-2014-JMS4195 - JMS 1059
EXPNO 11
PROCNO 1
Date_ 20141120
Time 14.58
INSTRUM AVIII400
PROBHD 5 mm PABBO BB-
PULPROG zgpg30
TD 65536
SOLVENT MeOD
NS 64
DS 4
SWH 96153.844 Hz
FIDRES 1.467191 Hz
AQ 0.3408372 sec
RG 2050
DW 5.200 usec
DE 6.50 usec
TE 293.2 K
D1 2.00000000 sec
D11 0.03000000 sec
TD0 1
======== CHANNEL f1 ========
NUC1 31P
P1 9.40 usec
PL1 0.00 dB
PL1W 23.83780289 W
SFO1 161.9310633 MHz
======== CHANNEL f2 ========
CPDPRG2 waltz16
NUC2 1H
PCPD2 80.00 usec
PL2 -1.00 dB
PL12 15.55 dB
PL13 19.00 dB
PL2W 12.26963711 W
PL12W 0.27153867 W
PL13W 0.12269637 W
SFO2 400.0416002 MHz
SI 32768
SF 161.9391600 MHz
WDW EM
SSB 0
LB 1.00 Hz
GB 0
PC 1.40
80
60
40
20
0
−20
−40
−60
−80
ppm
1.00
1.01

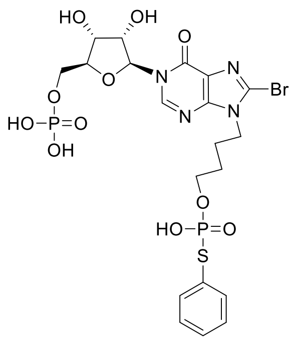
HO
OH
O
N
N
Br
O
N
N
HO−P=O
OH
O
HO−P=O
S

JMS 1062 TEA salt

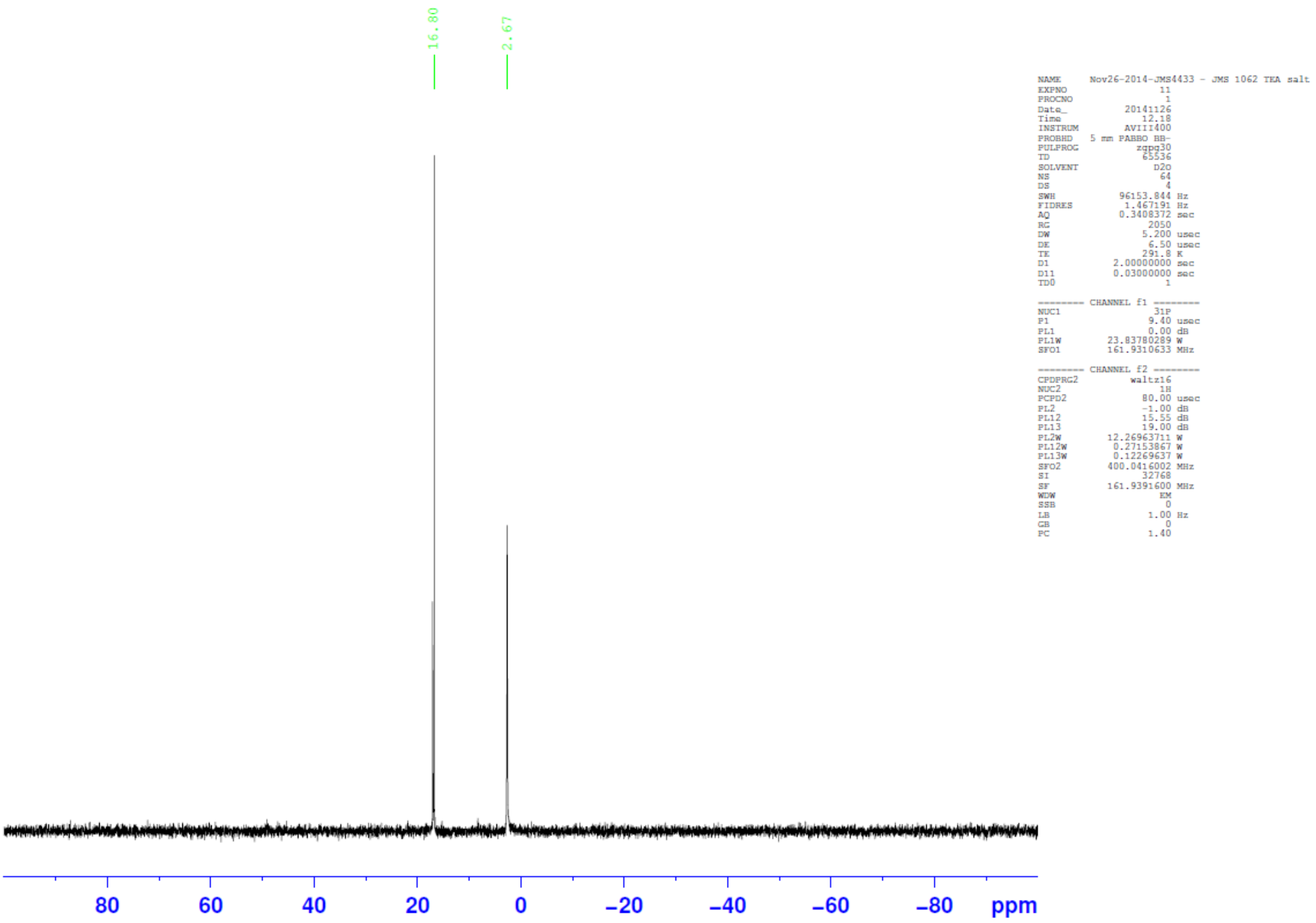

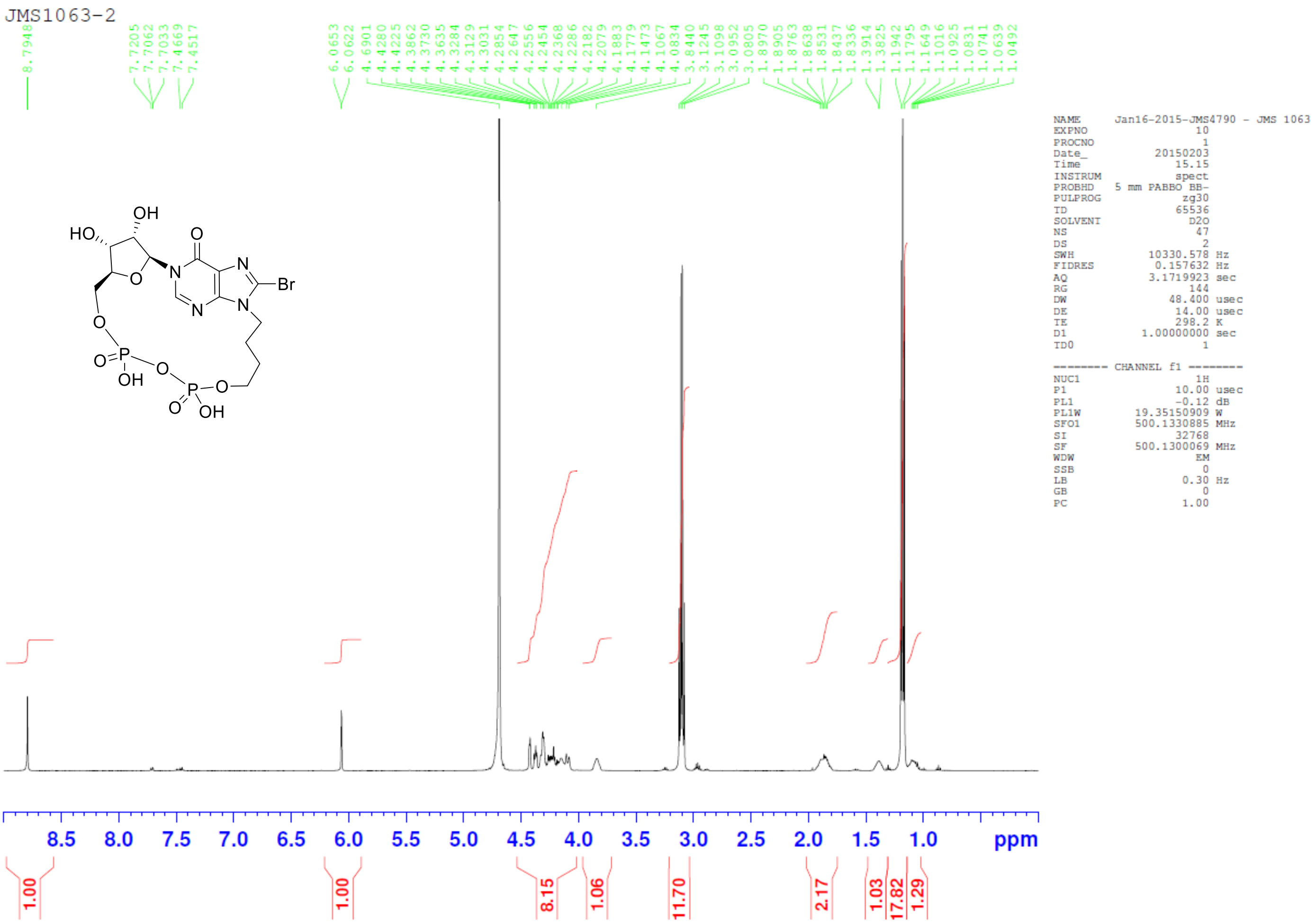
JMS1063-2
HO
OH
O
N
N
Br
O
O
N
N
O
O=P
OH
O
P
O
O
OH
NAME Jan16-2015-JMS4790 - JMS 1063
EXPNO 10
PROCNO 1
Date_ 20150203
Time 15.15
INSTRUM spect
PROBHD 5 mm PABBO BB-
PULPROG zg30
TD 65536
SOLVENT D2O
NS 47
DS 2
SWH 10330.578 Hz
FIDRES 0.157632 Hz
AQ 3.1719923 sec
RG 144
DW 48.400 usec
DE 14.00 usec
TE 298.2 K
D1 1.00000000 sec
TD0 1
======== CHANNEL f1 ========
NUC1 1H
P1 10.00 usec
PL1 -0.12 dB
PL1W 19.35150909 W
SFO1 500.1330885 MHz
SI 32768
SF 500.1300069 MHz
WDW EM
SSB 0
LB 0.30 Hz
GB 0
PC 1.00
8.5
8.0
7.5
7.0
6.5
6.0
5.5
5.0
4.5
4.0
3.5
3.0
2.5
2.0
1.5
1.0
ppm

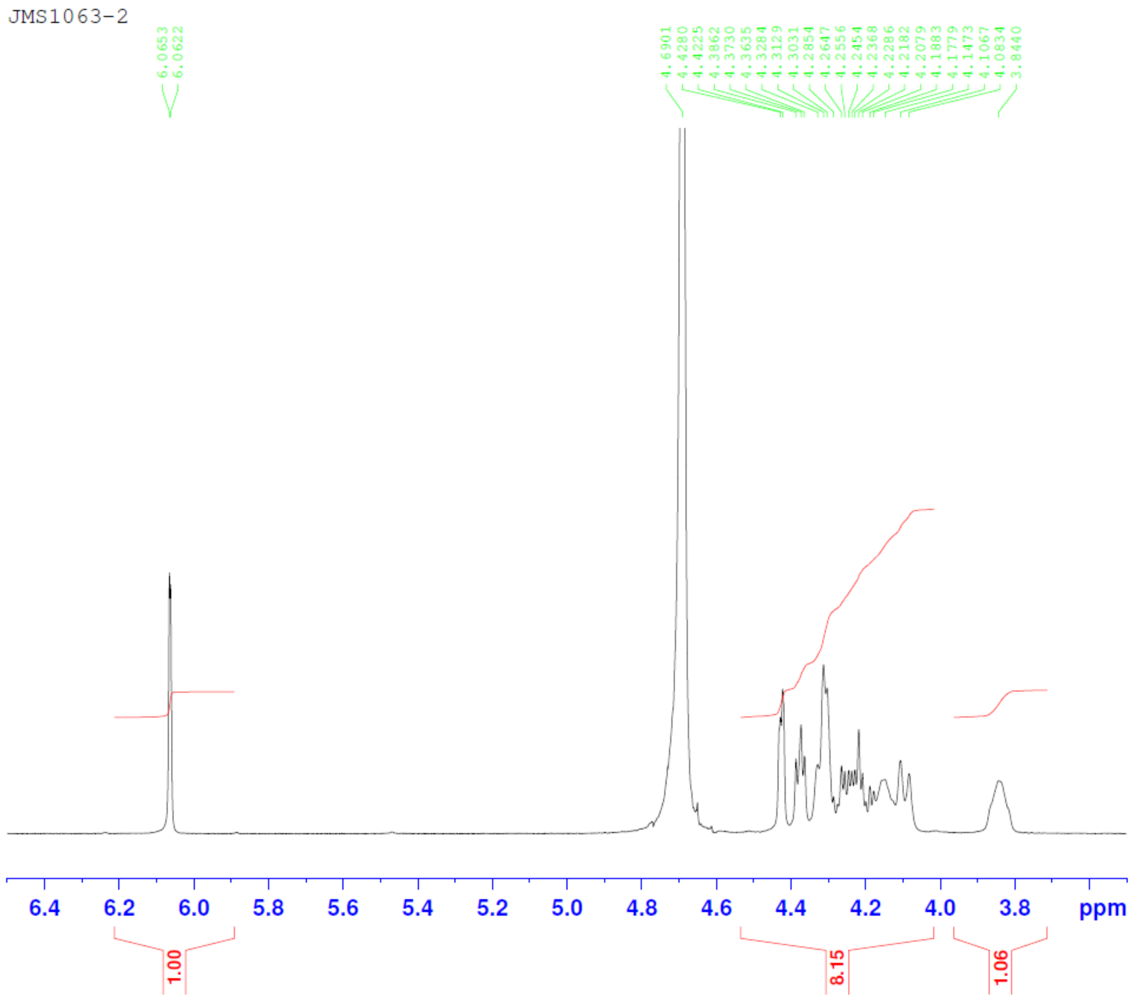
JMS1063-2
6.0653
6.0622
4.6901
4.4280
4.4225
4.3862
4.3730
4.3635
4.3284
4.3129
4.3031
4.2854
4.2647
4.2556
4.2454
4.2368
4.2286
4.2182
4.2079
4.1883
4.1779
4.1473
4.1067
4.0834
3.8440
6.4
6.2
6.0
5.8
5.6
5.4
5.2
5.0
4.8
4.6
4.4
4.2
4.0
3.8
ppm
1.00
8.15
1.06

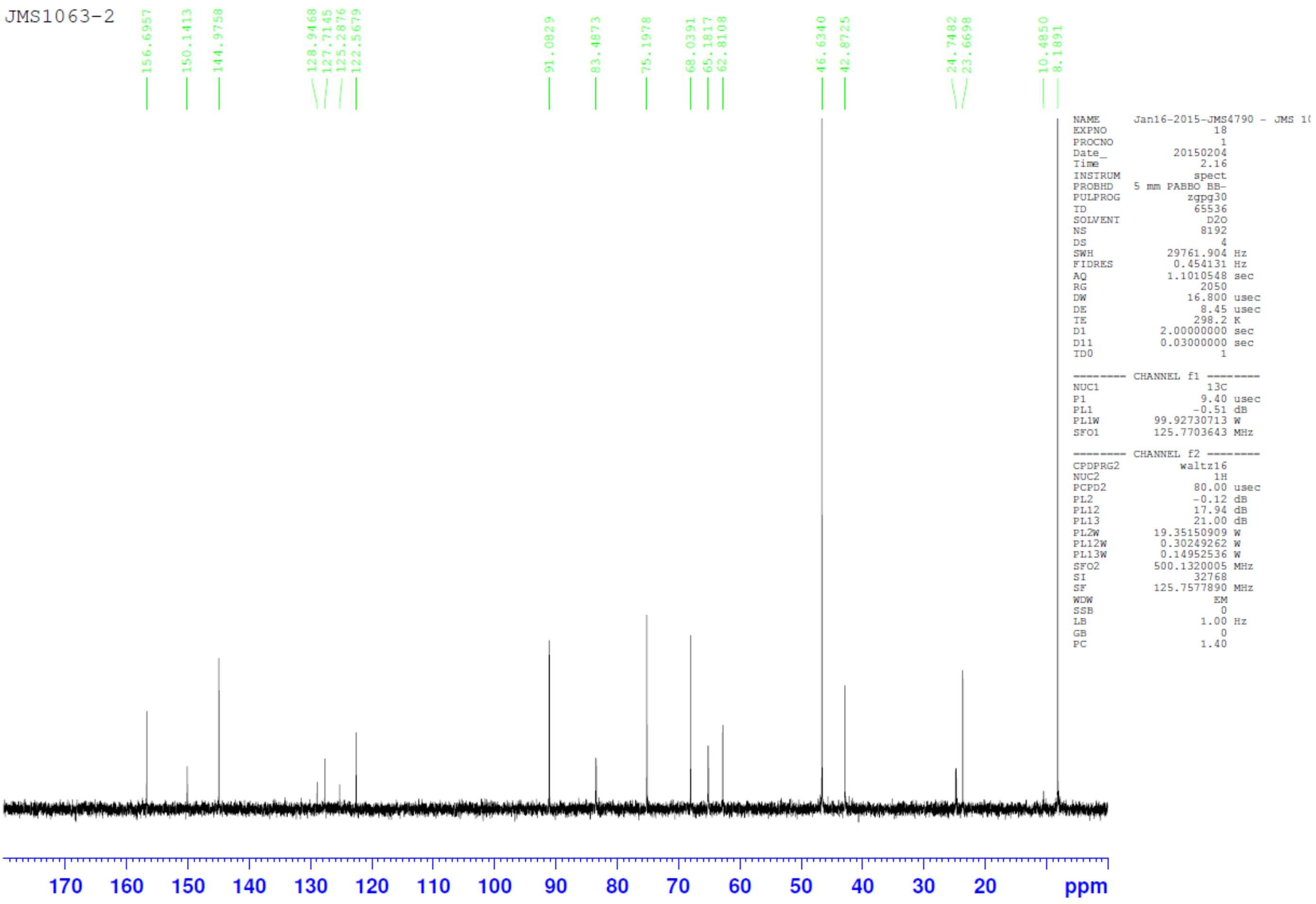
JMS1063-2
156.6957
150.1413
144.9758
128.9468
127.7145
125.2876
122.5679
91.0829
83.4873
75.1978
68.0391
65.1817
62.8108
46.6340
42.8725
24.7482
23.6698
10.4850
8.1891
NAME Jan16-2015-JMS4790 - JMS 1(
EXPNO 18
PROCNO 1
Date_ 20150204
Time 2.16
INSTRUM spect
PROBHD 5 mm PABBO BB-
PULPROG zgpg30
TD 65536
SOLVENT D2O
NS 8192
DS 4
SWH 29761.904 Hz
FIDRES 0.454131 Hz
AQ 1.1010548 sec
RG 2050
DW 16.800 usec
DE 8.45 usec
TE 298.2 K
D1 2.00000000 sec
D11 0.03000000 sec
TD0 1
======== CHANNEL f1 ========
NUC1 13C
P1 9.40 usec
PL1 -0.51 dB
PL1W 99.92730713 W
SFO1 125.7703643 MHz
======== CHANNEL f2 ========
CPDPRG2 waltz16
NUC2 1H
PCPD2 80.00 usec
PL2 -0.12 dB
PL12 17.94 dB
PL13 21.00 dB
PL2W 19.35150909 W
PL12W 0.30249262 W
PL13W 0.14952536 W
SFO2 500.1320005 MHz
SI 32768
SF 125.7577890 MHz
WDW EM
SSB 0
LB 1.00 Hz
GB 0
PC 1.40
170 160 150 140 130 120 110 100 90 80 70 60 50 40 30 20 ppm

JMS 1063 Ion Exchange

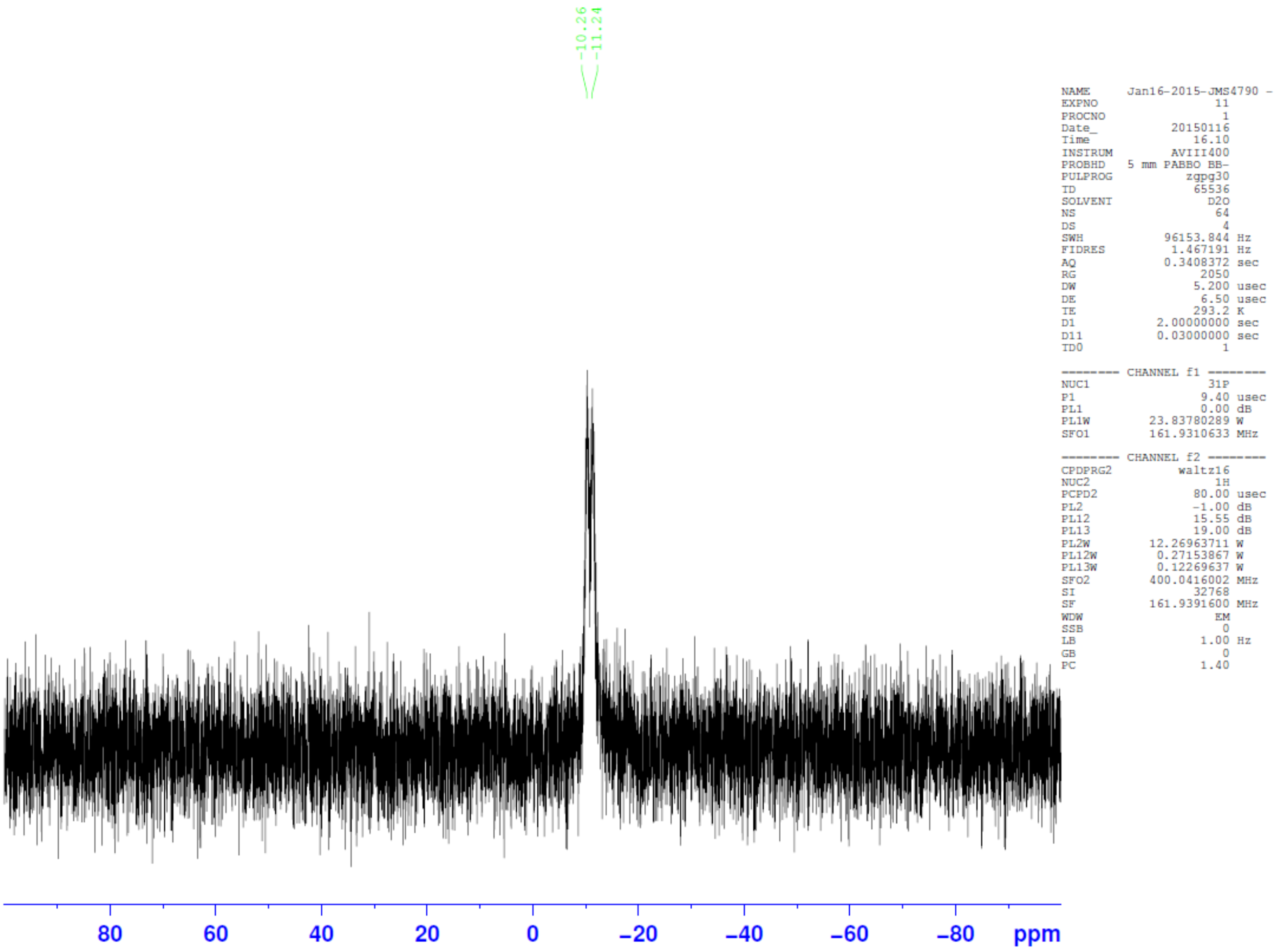


```
NAME       Jan16-2015-JMS4790 - JMS 1063
EXPNO                    11
PROCNO                    1
Date_              20150116
Time                  16.10
INSTRUM            AVIII400
PROBHD     5 mm PABBO BB-
PULPROG             zgpg30
TD                   65536
SOLVENT                D2O
NS                      64
DS                       4
SWH              96153.844 Hz
FIDRES            1.467191 Hz
AQ               0.3408372 sec
RG                    2050
DW                   5.200 usec
DE                    6.50 usec
TE                   293.2 K
D1              2.00000000 sec
D11             0.03000000 sec
TD0                      1

======== CHANNEL f1 ========
NUC1                   31P
P1                    9.40 usec
PL1                   0.00 dB
PL1W           23.83780289 W
SFO1           161.9310633 MHz

======== CHANNEL f2 ========
CPDPRG2            waltz16
NUC2                    1H
PCPD2                80.00 usec
PL2                  -1.00 dB
PL12                 15.55 dB
PL13                 19.00 dB
PL2W           12.26963711 W
PL12W           0.27153867 W
PL13W           0.12269637 W
SFO2           400.0416002 MHz
SI                   32768
SF             161.9391600 MHz
WDW                     EM
SSB                      0
LB                    1.00 Hz
GB                       0
PC                    1.40
```
